# The *PSEN1*, p.E318G Variant Increases the Risk of Alzheimer's Disease in *APOE*-ε4 Carriers

**DOI:** 10.1371/journal.pgen.1003685

**Published:** 2013-08-22

**Authors:** Bruno A. Benitez, Celeste M. Karch, Yefei Cai, Sheng Chih Jin, Breanna Cooper, David Carrell, Sarah Bertelsen, Lori Chibnik, Julie A. Schneider, David A. Bennett, Alzheimer's Disease Neuroimaging Initiative (ADNI), Genetic and Environmental Risk for Alzheimer's Disease Consortium (GERAD1), Anne M. Fagan, David Holtzman, John C. Morris, Alison M. Goate, Carlos Cruchaga

**Affiliations:** 1 Department of Psychiatry, School of Medicine, Washington University, St. Louis, Missouri, United States of America; 2 Program in Translational NeuroPsychiatric Genomics, Institute for the Neurosciences Department of Neurology, Brigham and Women's Hospital, Boston, Massachusetts, United States of America; 3 Harvard Medical School, Boston, Massachusetts, United States of America; 4 Program in Medical and Population Genetics, Broad Institute of Harvard University and M.I.T., Cambridge, Massachusetts, United States of America; 5 Rush Alzheimer's Disease Center and Department of Neurological Sciences, Rush University Medical Center, Chicago, Illinois, United States of America; 6 Department of Neurology, School of Medicine, Washington University, St. Louis, Missouri, United States of America; 7 Hope Center Program on Protein Aggregation and Neurodegeneration, Washington University St. Louis, Missouri, United States of America; 8 Department of Genetics, School of Medicine, Washington University, St. Louis, Missouri, United States of America; University of Miami, Miller School of Medicine, United States of America

## Abstract

The primary constituents of plaques (Aβ42/Aβ40) and neurofibrillary tangles (tau and phosphorylated forms of tau [ptau]) are the current leading diagnostic and prognostic cerebrospinal fluid (CSF) biomarkers for AD. In this study, we performed deep sequencing of *APP, PSEN1, PSEN2, GRN, APOE* and *MAPT* genes in individuals with extreme CSF Aβ42, tau, or ptau levels. One known pathogenic mutation (*PSEN1* p.A426P), four high-risk variants for AD (*APOE* p.L46P, *MAPT* p.A152T, *PSEN2* p.R62H and p.R71W) and nine novel variants were identified. Surprisingly, a coding variant in *PSEN1*, p.E318G (rs17125721-G) exhibited a significant association with high CSF tau (p = 9.2×10^−4^) and ptau (p = 1.8×10^−3^) levels. The association of the p.E318G variant with Aβ deposition was observed in *APOE*-ε4 allele carriers. Furthermore, we found that in a large case-control series (n = 5,161) individuals who are *APOE*-ε4 carriers and carry the p.E318G variant are at a risk of developing AD (OR = 10.7, 95% CI = 4.7–24.6) that is similar to *APOE*-ε4 homozygous (OR = 9.9, 95% CI = 7.2.9–13.6), and double the risk for *APOE*-ε4 carriers that do not carry p.E318G (OR = 3.9, 95% CI = 3.4–4.4). The p.E318G variant is present in 5.3% (n = 30) of the families from a large clinical series of LOAD families (n = 565) and exhibited a higher frequency in familial LOAD (MAF = 2.5%) than in sporadic LOAD (MAF = 1.6%) (p = 0.02). Additionally, we found that in the presence of at least one *APOE*-ε4 allele, p.E318G is associated with more Aβ plaques and faster cognitive decline. We demonstrate that the effect of *PSEN1*, p.E318G on AD susceptibility is largely dependent on an interaction with *APOE*-ε4 and mediated by an increased burden of Aβ deposition.

## Author Summary

Alzheimer's disease (AD) is the most common neurodegenerative disease affecting more than 5.3 million people in the US. AD-causing mutations have been identified in *APP*, *PSEN1* and *PSEN2* genes. Heterozygous carriers of *APOE*-ε4 allele exhibit a 3-fold increased risk for developing AD, while homozygous carriers show a 10-fold greater risk than non-carriers. Here, we sequenced individuals with extreme levels of well-established AD cerebrospinal fluid (CSF) biomarkers in order to identify variants in *APOE, APP, PSEN1, PSEN2, GRN* and *MAPT* genes associated with AD risk. This approach allowed us to identify known pathogenic variants, additional AD risk genetic factors and identify a low frequency variant in *PSEN1*, p.E318G (rs17125721-G) that increases risk for AD in a gene-gene interaction with *APOE*. These findings were replicated in three large (>4,000 individuals) and independent datasets. This finding is particularly important because we demonstrated that a currently considered non-pathogenic variant is associated with higher levels of neuronal degeneration, and with Aβ deposition, more Aβ plaques and faster cognitive decline in an *APOE*-ε4-dependent fashion. *APOE*-ε4 heterozygous individuals who carry this variant are at similar AD risk as *APOE*-ε4 homozygous individuals.

## Introduction

Dementias are complex, polygenic and genetically heterogeneous disorders [1]. The most common form of dementia is Alzheimer's disease (AD), which affects more than 5.3 million people in the US [2]. Late-onset AD (LOAD) is the most common form of dementia. However, the current model of AD pathogenesis is based on the genetic findings in rare and phenotypically extreme AD cases [3]. LOAD heritability varies from 58% to 79% [4] and, despite the tremendous progress in AD genetics in the last twenty years, the total proportion of phenotypic variance explained by all the combined variants (including *APOE* genotype and genome wide association studies [GWAS] signals) is estimated to be 23% [5], which suggests a large proportion of the heritability of AD still remains unexplained. Three important factors may account for the missing heritability in AD; first, the clinical heterogeneity of AD remains a significant confounding variable in case-control studies [6], second, much of the unexplained variance of complex phenotypes may be attributed to low frequency or rare alleles [7] and third, gene by gene or gene by environment interactions [8]. Quantitative intermediate phenotypes have helped to overcome some of these obstacles in complex diseases [9], [10]. Endophenotype-oriented approaches have greater statistical power, less clinical heterogeneity and offer important insights into the mechanisms by which genetic variants modulate the disease phenotype [6], [9], .

The primary constituents of plaques (Aβ42/Aβ40) and neurofibrillary tangles (tau and phosphorylated forms of tau [ptau]) are the current leading diagnostic and prognostic cerebrospinal fluid (CSF) biomarkers for AD [12]. Recently, it was shown that CSF biomarker abnormalities typically precede clinical AD symptoms by decades and reflect the timing and magnitude of pathophysiological changes [13]. These findings suggest that a better understanding of the genetic contribution to the variance in these CSF biomarkers can provide important information about susceptibility to AD. In fact, the two most important known risk factors for AD, *APOE* genotype and age account for 13% and 14% of the variance in CSF Aβ42 and tau levels, respectively [14]. Likewise, pathogenic mutations in the most important causal genes for familial AD, *amyloid-beta precursor protein* (*APP*), and *presenilin 1* and 2 (*PSEN1, PSEN2*) alter CSF Aβ42 levels [13], [15], [16]. Additionally, some genetic variants initially discovered by their association with CSF biomarkers have recently been proven to be modifiers of risk, age at onset (AAO) or rate of AD progression [17], [18], [19]. Likewise, it was recently described that carriers of *PSEN1* mutations exhibit very low CSF Aβ42, and high tau or ptau levels [13], [20], [21], [22]. Similar CSF biomarker level profiles have been described in sporadic AD cases [23]. However, the genetic variants responsible for CSF changes in sporadic AD have not been found yet. Together, these results suggest that CSF biomarker levels as quantitative traits are useful tools in uncovering genetic variants that are closely related to the physiopathological mechanisms underlying AD.

Rare or low frequency coding and non-coding variants have been predicted to be enriched in functional alleles and to exhibit strong effect size [7], [10]. Recently, a rare (minor allele frequency [MAF] = 0.02) coding variant in *TREM2* gene p.P47H was found to confer a high risk for AD (Odd ratios from 2 to 5) [24], [25], [26]. Two recent studies analyzed the association of genetic variants of *APP, PSEN1, PSEN2, MAPT, and GRN* on risk for AD [27], [28]. One study was focused on common variants in sporadic AD [27] while the other focused on the identification of very rare coding variants in familial LOAD [28]. However, the impact of low-frequency coding variants of *APP, PSEN1*, *PSEN2, GRN* and *MAPT* on sporadic LOAD has not been well studied. Identification of low frequency variants associated with disease remains challenging because standard case-controls design requires very large sample sizes. To overcome this problem we have used quantitative phenotypes. Previously, we identified a pathogenic mutation in a family with LOAD within the *PSEN1* gene by selecting the top and bottom 5% from the distributions of Aβ40, Aβ42, and Aβ42/40 ratio [29] In the present study, we sequenced individuals with extremes levels of CSF-based biomarkers in order to identify variants in *APOE, APP, PSEN1*, *PSEN2, GRN* and *MAPT* genes associated with the CSF biomarker levels. This approach allowed us to identify known pathogenic variants, AD risk factors and identify a low frequency variant that increases risk for AD in a gene-gene interaction mode.

## Results

### Rare variants found by targeted-pooled-DNA and Next Generation sequencing

We hypothesized that the coding variants found in individuals at the extremes of the phenotypic distribution of CSF biomarker levels are more likely to have a functional impact on CSF biomarker levels. In order to identify rare or low frequency variants that affect the CSF levels of Aβ42, tau and ptau levels, we used a two-stage extreme phenotype sequencing design (Figure S1). A 10-fold difference between the lowest and highest raw values in Aβ42, tau and ptau CSF levels in each series was found among individuals in these studies. The individuals were selected regardless of their clinical status (based on the clinical dementia rating [CDR]) (Table 1). We combined both series (WU-ADRC [n = 475] and ADNI [n = 259]) by normalizing the CSF Aβ42, tau and ptau levels and adjusting for covariates [17], [18]. We selected 212 individuals from the top and bottom 15% for each phenotype (Table 1). The 212 samples were divided in two pools (Pool 1 and 2, respectively); targeted and pooled-sample sequencing was performed. All the validated variants were genotyped in the total CSF sample and tested for association with each CSF biomarker. Linear regression (assuming an additive genetic effect) was utilized for each variant by adjusting for significant covariates (age, gender, CDR and site [WU-ADRC or ADNI]) (Table S1 in Text S1) [17], [18].

**Table 1:** Summary of sample characteristics.

	WU-ADRC	ADNI
**Total CSF Samples:**		
**N**	475	259
**Age (years)** **Mean ± SD (range)**	69±10 (37–91)	76±7 (56–91)
**APOE ε4+ (%)**	39	47
**CDR 0 (%)**	73	40
**Aβ42 Low (%)**	44	66
**Female (%)**	60	39
**ptau**	54 (18–237)	29.5 (8–113)
**Tau**	283 (86–1303)	82 (32–327.5)
**Aß_42_**	551 (165–1412)	155 (71–300)
**Pool 1:**		
**N**	70	28
**Age (years)** **Mean ± SD (range)**	70±10 (50–91)	76±7 (56–87)
**APOE ε4+ (%)**	43	39
**CDR 0 (%)**	77	39
**Aβ42 Low (%)**	24	75
**Female (%)**	59	43
**ptau**	46 (20–199)	17 (10–63)
**Tau**	256 (93–713)	52 (32–135)
**Aß_42_**	757 (241–1412)	140 (81–256)
**Pool 2:**		
**N**	75	39
**Age (years)** **Mean ± SD (range)**	69±10 (46–91)	76±7 (60–90)
**APOE ε4+ (%)**	35	28
**CDR 0 (%)**	73	33
**Aβ42 Low (%)**	74	26
**Female (%)**	70	36
**ptau**	77 (24–237)	52 (8–133)
**Tau**	410 (90–1303)	120 (36–301)
**Aß_42_**	337 (175–1156)	157 (90–300)
**Additional Set:**		
**N**	330	192
**Age (years)** **Mean ± SD (range)**	67±11 (37–89)	76±7 (57–91)
**APOE ε4+ (%)**	42	53
**CDR 0 (%)**	73	42
**Aβ42 Low (%)**	41	67
**Female (%)**	58	46
**ptau**	54 (18–229)	30 (11–81)
**Tau**	273 (86–1204)	86 (24–328)
**Case-Control Set**	**Cases**	**Control**
**N**	1031	824
**Age (years)** **Mean ± SD (range)**	72.8±8.8(44–103)	77.7±8.8(53–105)
**APOE ε4+ (%)**	0.61	0.23
**CDR 0 (%)**	0	100
**Female (%)**	48	47

Age at lumbar puncture (LP), percentage of females, percentage of *APOE4* allele carriers, clinical dementia rating (CDR) at LP date for each sample and percentage of individuals with Low (L) levels of Aβ42 normalized for each site (see methods). For each phenotype the median in pg/ml and the range is shown. Charles F. and Joanne Knight Alzheimer's Disease Research Center at University of Washington (WU-ADRC) and Alzheimer's Disease Neuroimaging Initiative (ADNI). Cerebrospinal Fluid (CSF).

A greater than 30-fold coverage per allele at all positions within the 62 amplicons designed to cover the protein coding regions of the *APP, APOE, PSEN1, PSEN2, MAPT* and *GRN* were obtained (Table S2 in Text S1). After adjusting for the sensitivity and specificity parameters of the base-calling algorithm (SPLINTER) using negative and positive controls, a total of 396 and 369 variants were called and perfectly annotated in the targeted genomic regions of Pool 1 and 2, respectively. 73% of these variants were intronic, 8% were missense, 5% were coding-synonymous, 1% were at splicing sites, 12% were located at the untranslated regions (UTR) and 2% were called to be near-gene (Table S2b in Text S1) We focused on missense and splicing-affecting variants with a predicted minor allele frequency (MAF) below 5% (by SPLINTER) in each pool.

A total of 27 rare or low frequency non-synonymous variants were validated by direct genotyping in the discovery samples (both pools). 33% of these variants identified (9/27) were novel. Seven of nine (77%) are located in highly conserved nucleotide (GERP>4) and 88% (8/9) are predicted to be damaging for the respective protein (SIFT and polyphen2 algorithms) [30]. As expected, 48% of these variants are singletons (9/27) or doubletons (4/27) (Table 2).

**Table 2:** Summary of the rare variants found in the extreme values of CSF biomarker levels.

Gene	AA Substitution	dbSNP ID	GERP Score	Protein prediction	MAF in ESV	Total #Hets	Total MAF	Clinical Interpretation
**APOE**	E37K	rs142480126	−6.31	TOLERATED/benign	0.00008	1	0.0005	
	L46P	rs769452	−5.71	TOLERATED/possibly damaging	0.0010	6	0.003	Pathogenic nature unclear
**APP**	A741S	Novel	6.06	DAMAGING/probably damaging	0	3	0.0016	
	V287G	Novel	3.62	DAMAGING/possibly damaging	0	3	0.0016	
**GRN**	R433W	rs63750412	1.53	DAMAGING/possibly damaging	0.002	9	0.005	Not pathogenic
	P458L	rs63750537	4.32	TOLERATED/probably damaging	0	1	0.0005	Not pathogenic
	R19W	rs63750723	2.66	TOLERATED/benign	0.016	5	0.003	Not pathogenic
	C231W	rs117758963 §	4.13	DAMAGING/probably damaging	0	13	0.007	
	C247Y	Novel	4.81	DAMAGING/probably damaging	0	1	0.0005	
**MAPT**	T263P*	Novel	5.41	DAMAGING/probably damaging	0	1	0.0005	
	G107S	rs144397565	4.62	TOLERATED/probably damaging	0.0005	1	0.0005	
	S318L	rs73314997	4.38	TOLERATED/benign	0.06	16	0.01	
	V224G	rs141120474	5.46	TOLERATED/possibly damaging	0.003	9	0.005	
	Q230R	rs63750072	4.14	TOLERATED/probably damaging	0.04	100	0.05	Not pathogenic
	A152T*	rs143624519	3.15	TOLERATED/benign	0.002	5	0.003	Pathogenic nature unclear
**PSEN1**	A426P	rs63751223	4.37	TOLERATED/probably damaging	0	1	0.0005	Pathogenic
	R35Q	rs63750592	3.09	TOLERATED/benign	0.0004	1	0.001	Not pathogenic
	V63G	Novel	5.15	TOLERATED/benign	0	2	0.001	
	E318G	rs17125721	5.53	DAMAGING/benign	0.014	32	0.017	Not pathogenic
**PSEN2**	G270S	Novel	3.38	DAMAGING/possibly damaging	0	1	0.0005	
	E317G	rs78420366 §	4.8	TOLERATED/benign	0	3	0.0016	
	A346S	Novel	5.16	TOLERATED/possibly damaging	0	2	0.0011	
	T347P	Novel	5.16	TOLERATED/probably damaging	0	2	0.0011	
	T369S	Novel	5.68	DAMAGING/probably damaging	0	2	0.0011	
	R62H	rs58973334	−4.6	TOLERATED/benign	0.01	9	0.005	Pathogenic nature unclear
	R71W	rs140501902	2.92	DAMAGING/benign	0.003	5	0.003	Pathogenic nature unclear
	V300G	rs77421307 §	4.95	DAMAGING/possibly damaging	0	1	0.0005	

Gene: official Symbol provide by HGNC; dbSNP: variants with or without rs numbers. AA Substitution: amino acid change resulting from the observed variant; dbSNP ID: rs# for variants present in dbSNP 135, Novel for variants not present in dbsnp, 1000 genome or Exome Variant Server; GERP score: Genomic Evolutionary Rate Profiling score; Protein prediction: based on SIFT/Polyphen2 analysis of the predicted effect of the substitution on protein function; MAF in ESV: Minor allele frequency in Exome Variant Server; Total # Hets: Number of carriers of the variant in the total sample; Total MAF: Minor allele frequency in all sample genotyped. Clinical Interpretation: Clinical interpretation is based on AD&FTD mutation database and published papers.

§ dbSNP 135 appears as validation pending

Among the 18 previously reported variants; we found one known pathogenic mutation *PSEN1* p.A426P. *PSEN1* p.A426P (rs63751223) was reported in a five members of a family with autosomal dominant AD [31].We also found four high-risk variants for LOAD (*APOE*, p.L46P; *MAPT*, p.A152T; *PSEN2*, p.R62H and p.R71W) [28], [32], [33], six variants that were previously reported in families with AD or frontotemporal dementia (FTD), but classified as non-pathogenic (*GRN*, p.R433W, p.P458L, p.R19W; *MAPT*, p.Q230R; *PSEN1*, p.R35Q and p.E318G) [34], and seven variants that have been recently reported in public databases with no clear role in human disease to date (*APOE*, p.E37K; *GRN*, p.C231W; *MAPT*, p.G107S, p.S318L, p.V224G; *PSEN2*, p.E317G and p.V300G) (A detailed description of each variant can be found in the supporting material in Text S1).

These results highlight the relative enrichment of rare and low frequency variants in six genes involved in AD and FTD among individuals at the extremes of the CSF biomarker distribution [29].

### Association with CSF biomarker levels

**Figure 1:**
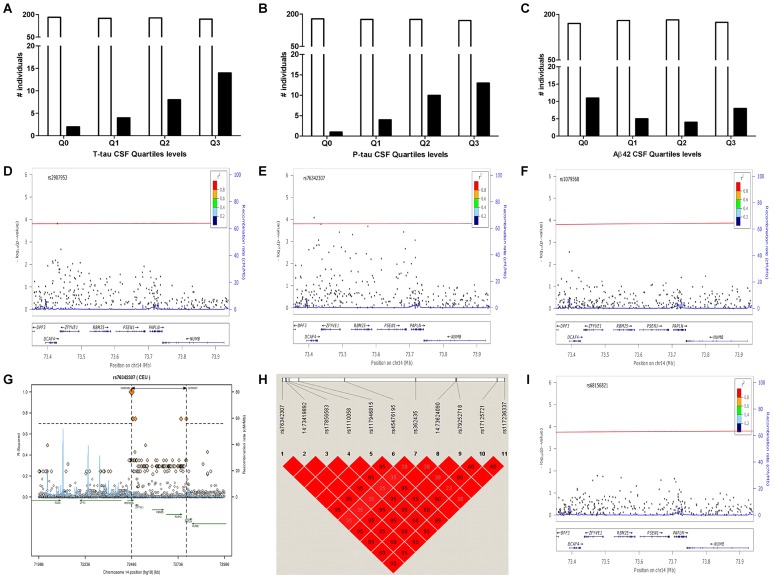
Distribution of *PSEN1* p.E318G mutation carriers in CSF Biomarker quartiles. **A**. CSF tau, Logistic regression model p = 6.0×10^−4^. **B**. CSF pTau, Logistic regression model p = 3.0×10^−4^. **C**. CSF Aβ42, Logistic regression model p = 0.38. White bars represent the number of non carriers. Black bars represent the number of carriers **D**. Association of *PSEN1* gene with CSF tau. **E**. Association of *PSEN1* gene with CSF ptau. **F**. Association of *PSEN1* gene with CSF Aβ42. Plots are showing the most significant SNP at a given locus along with the combined-analysis results for SNPs in the region surrounding it (typically ±500 kb). Symbols are colored according to the LD of the SNP with the top SNP (r^2^ color-based insert). The red line represents the threshold for significance. The light blue line represents the estimated recombination rate. **G**. LD Block for the most significant SNP associated with biomarker levels at *PSEN1* genomic region: SNP rs76342307 based on the 1000 genome project for Europeans. Gene annotations are shown as dark green lines. **H**. LD Block for rs76342307 and rs17125721 in our own data set. **I**. Plot after the conditional analysis including both SNPs (rs76342307 and rs17125721) in the model.

Next, we tested whether any of the variants identified by an endophenotype-based approach could improve our understanding of both the genetic architecture and pathophysiology of LOAD [17], [18]. We ran a linear regression analysis for single SNP using CSF biomarkers as quantitative traits, but we failed to find significant association with CSF tau, ptau or Aβ42 levels for most of the identified variants, even after we collapsed all of the potentially damaging variants in each gene and analyzed the dataset for carriers vs. non-carriers of these variants (Table 3). Surprisingly, a low frequency coding variant in *PSEN1*, p.E318G (rs17125721) (MAF = 0.02 for Europeans Americans, Exome Variant Server EVS: http://evs.gs.washington.edu/EVS/), whose pathogenic role is currently debated [34] exhibited a statistically significant association (multiple test correction threshold, p = 7.0×10^−3^) with CSF tau (p = 9.2×10^−4^, Beta = 0.14) and ptau levels (P = 1.8×10^−3^, Beta = 0.12), but not with Aβ42 (p = 0.14, Beta = −0.05). Interestingly, it has been reported that the combination of Aβ42 and tau or ptau as a ratio provides the best discriminative value to date for AD cases [35], [36] and predict the conversion from non-dementia clinical status to dementia [37]. p.E318G exhibited a significant association with the ratio of ptau∶Aβ42 (p = 9.5×10^−5^, Beta = 0.08) and tau∶Aβ42 (p = 2.0×10^−4^, Beta = 0.06) (Figure 1A–C, 2A) suggesting that the association of p.E318G with CSF biomarker levels may be an association with clinical AD.

**Figure 2:**
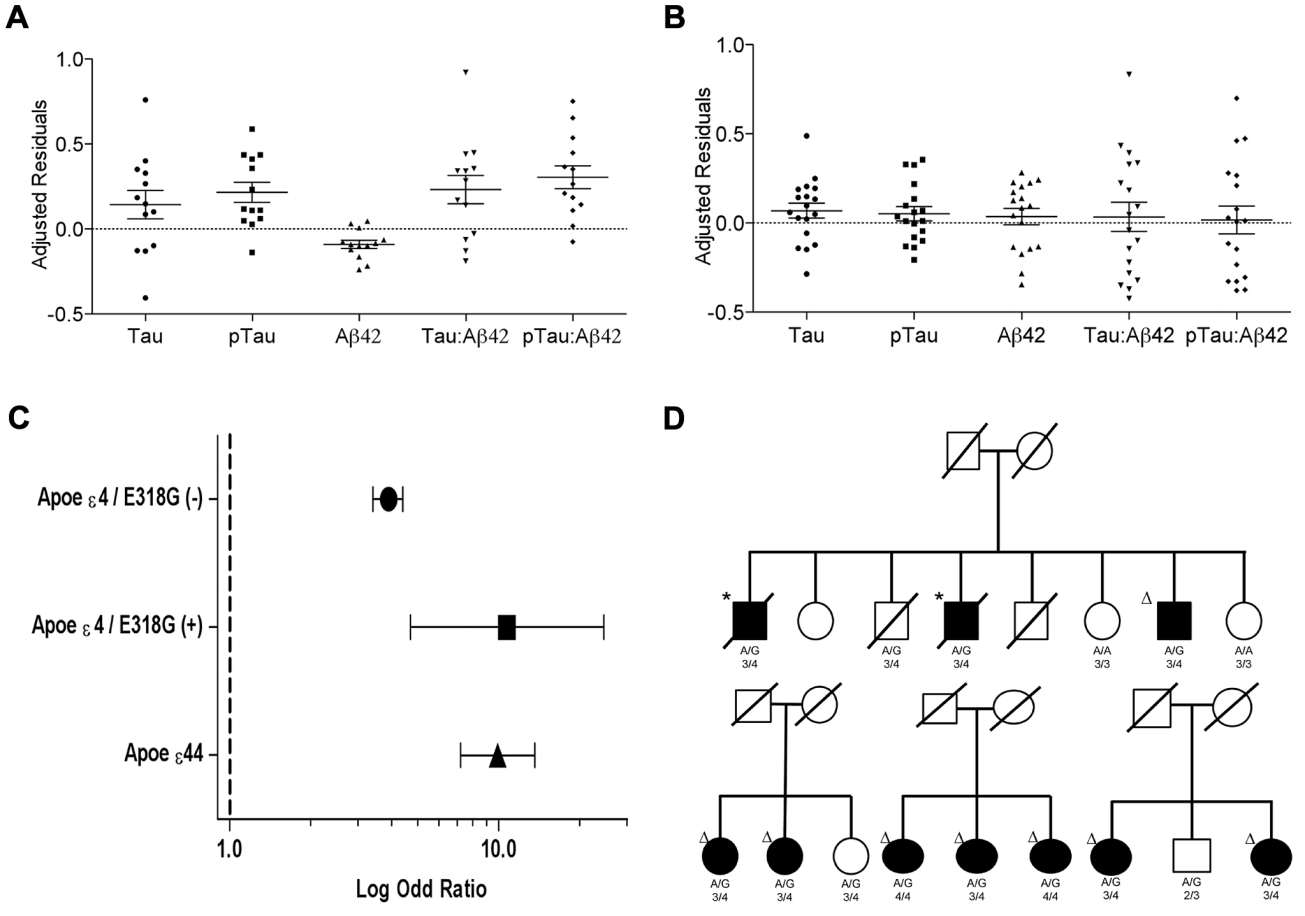
Distribution of biomarker levels in *PSEN1* p.E318G and *APOE* carriers. Distribution of the covariate-adjusted residuals of the CSF tau, ptau, Aβ42, Tau∶Aβ42 and ptau∶Aβ42 ratio. **A**. *APOE* ε4 positive/*PSEN1* p.E318G carriers and. **B**. *APOE* ε4 negative/*PSEN1* p.E318G carriers. **C**. Forest plot of the odd ratios of the effect of *PSEN1* p.E318G in *APOE* ε4 heterozygous. **D**. Pedigrees for some of the families with p.E318G carriers illustrating the segregation analysis and the presence of *APOE* ε4 allele. A/G or A/A is the genotype for p.E318G variant. 2/3, 3/3, 3/4, 4/4 is the APOE genotype. * Symbol means confirmed AD by autopsy. Δ Symbol indicates probable AD diagnosed using NINCDS-ADRDA criteria.

**Table 3:** Frequency of the rare variants in cases and controls and in individuals with and without Aβ deposition.

Gene	AA	Clinical status					Status by Aβ deposition				
		MAF CDR = 0 (n = 667)	# Het	MAF CDR>0 (n = 258)	Het	p value	MAF controls (High Aβ42, n = 427)	# Het	MAF in cases (Low Aβ42, n = 500)	# Het	p value
**APOE**	E37K	0.0007	1	0	0	1.00	0.001	1	0	0	0.46
	L46P	0.0030	4	0.004	2	1.00	0.004	3	0.003	3	1.00
	**Total**	**0.004**	**5**	**0.004**	**2**	**0.21**	**0.005**	**4**	**0.003**	**3**	**0.82**
**APP**	A741S	0.0007	1	0.0039	2	0.48	0.0012	1	0.002	2	1.00
	V287G	0.0022	3	0	0	1.00	0.0035	3	0	0	0.10
	**Total**	**0.003**	**4**	**0.004**	**2**	**0.67**	**0.005**	**4**	**0.002**	**2**	**0.69**
**GRN**	R433W	0.0045	6	0.0058	3	0.71	0.0047	4	0.005	5	0.79
	P458L	0	0	0.0019	1	0.28	0	0	0.001	1	1.00
	R19W	0.0022	3	0.0039	2	0.67	0.0023	2	0.003	3	0.66
	C231W	0.0060	8	0.0096	5	0.19	0.007	6	0.007	7	1.00
	C247Y	0.0007	1	0	0	1.00	0	0	0.001	1	1.00
	**Total**	**0.013**	**18**	**0.021**	**11**	**0.21**	**0.014**	**12**	**0.017**	**17**	**0.18**
**MAPT**	T263P*	0.0007	1	0	0	1.00	0	0	0.001	1	1.00
	G107S	0.0007	1	0	0	1.00	0.0012	1	0	0	1.00
	S318L	0.0104	14	0.004	2	0.16	0.013	11	0.005	5	0.07
	V224G	0.0030	4	0.010	5	0.28	0.006	5	0.004	4	1.00
	Q230R	0.0471	66	0.062	34	0.15	0.049	44	0.053	56	0.67
	A152T*	0.0015	2	0.006	3	0.14	0.002	2	0.003	3	1.00
	**Total**	**0.063**	**87**	**0.083**	**45**	**0.09**	**0.07**	**62**	**0.067**	**70**	**0.85**
**PSEN1**	A426P	0.0007	1	0	0	0.28	0.001	1	0	0	0.46
	R35Q	0	0	0.0019	1	1.00	0.001	1	0	0	0.46
	V63G	0.0015	2	0	0	1.00	0.002	2	0	0	0.22
	E318G	0.0155	21	0.021	11	0.42	0.013	11	0.021	21	0.20
	**Total**	**0.018**	**25**	**0.021**	**11**	**0.78**	**0.017**	**15**	**0.021**	**21**	**0.86**
**PSEN2**	G270S	0	0	0.0019	1	0.28	0.0012	1	0	0	0.46
	E317G	0.001	1	0.0039	2	0.28	0.0023	2	0.001	1	0.60
	A346S	0.001	1	0.0019	1	0.48	0.0012	1	0.001	1	1.00
	T347P	0.001	2	0	0	1.00	0.0023	2	0	0	0.22
	T369S	0.001	2	0	0	1.00	0.0023	2	0	0	0.22
	R62H	0.005	7	0.0039	2	0.74	0.0047	4	0.005	5	1.00
	R71W	0.001	1	0.008	4	**0.03**	0.0012	1	0.004	4	**0.3**
	V300G	0.001	1	0	0	1.00	0.0012	1	0	0	0.46
	**Total**	**0.011**	**15**	**0.019**	**10**	**1.00**	**0.016**	**14**	**0.011**	**11**	**0.31**

Gene: official Symbol provide by HGNC; AA Substitution: amino acid change resulting from the observed variant; CDR at the time of the Lumbar punction. Samples were stratified based on the CSF Aβ42 levels as an approximation to the Aβ deposition. For ADNI-CSF series the cut-off was Aβ42 = 192 pg/mL and in WU-ADRC-CSF series we used a CSF Aβ42 = 500 pg/mL.

In order to confirm this association with CSF biomarkers and to determine whether this or any other SNP in linkage disequilibrium (LD) was driving the association, we combined genotype and imputed data from 895 individuals (WU-ADRC, n = 501, and ADNI, n = 394, this dataset constitute the same CSF series that we genotyped (Table 1) plus additional 161 individuals) to perform a dense fine mapping analysis of *PSEN1* genomic region. The number of independent tests (M_eff_ = 317) was calculated based on the number of SNPs after correcting for LD structure (r^2^ = 0.8) within the genomic region (250 Kb in each side) [38]. We performed linear regression assuming an additive genetic model to test the association between each SNP and CSF biomarker levels by adjusting for age, gender and the first three principal components from the population stratification analysis. We confirmed a significant association (multiple-testing threshold = 1.6×10^−4^) between an intronic SNP, rs76342307 (MAF = 0.016) and CSF ptau (p = 8.0×10^−5^, Beta = 0.14), tau (p = 8.4×10^−3^, Beta = 0.10), and Aβ42 levels (p = 0.02, Beta = −0.06) (Figure 1D–F) for the *PSEN1* genomic region. Rs76342307 is located 0.2 Mb 3′ upstream from the *PSEN1* gene. We used data from the HapMap and the 1000 Genomes Project to identify all of the SNPs in linkage disequilibrium (LD, r^2^>0.8) with rs76342307. Six SNPs (rs76342307, rs17856583, rs1110058, rs117946815, rs117236337 and rs2091912) were found to be in strong LD (r^2^ = 0.95, D′ = 1) with rs76342307 spanning 0.3 Mb (Figure 1G, H). 100% and 97% concordance rates were observed among the directly typed and imputed results for rs76342307 and rs117236337, respectively. Interestingly, rs117236337 is an intronic SNP in *PSEN1* gene, which is also associated with extreme CSF tau (p = 0.02, Beta = 0.08), ptau (p = 5.7×10^−4^, Beta = 0.09) and Aβ42 levels (p = 0.01, Beta = −0.06). Next, we tested whether *PSEN1*, p.E318G was in LD with the SNPs identified by the fine mapping analysis. In fact, rs17125721 (*PSEN1*, p.E318G) is in moderate LD with all of them (R^2^ = 0.68, D′ = 1) (Figure 1H). To analyze whether the p.E318G and rs76342307 are two independent signals, we ran a conditional analysis including both SNPs (rs76342307 and rs17125721) in the model. When one of the SNPs was included in the model, the association from the other SNP disappeared, suggesting that the association in this locus is driven by a single signal (Figure 1I).

### Effect of *PSEN1*, p.E318G on Aβ deposition is *APOE* ε4-dependent

We observed that in the subset of individuals with Aβ deposition (CSF Aβ42 levels lower than 500 pg/ml in WU-ADRC, and 192 pg/ml in ADNI) [35], [39], the frequency of p.E318G carriers (4.2%, 21/500) was higher than in individuals without Aβ deposition (2.5%, 11/427), although this difference did not achieve statistical significance (p = 0.18, OR = 1.6, 95%CI = 0.78–3.4) (Table 3, 4). In addition, we observed that 93% (15/16) of the individuals carrying *PSEN1*, p.E318G along with *APOE* ε4 exhibited low CSF Aβ42 levels, while only 45% (9/20) of the individuals carrying *PSEN1*, p.E318G but do not carry the *APOE* ε4 allele showed low CSF Aβ42 levels, suggesting that *APOE* ε4 allele is modifying the profile of Aβ deposition in *PSEN1*, p.E318G carriers (Table 4 and Figure 2A). APOE ε4 is strongly associated with CSF Aβ42 levels (Table 4) [14], [18], and *APOE* genotype has been reported to modify disease expression in individuals with mutations in *PSEN1* [40] and *PSEN2* [41] genes. However, previous reports have not found any significant interaction between *APOE* and *PSEN1* p.E318G, most likely due to the low frequency of *PSEN1*, p.E318G and small sample sizes [42], [43], [44]. To analyze whether there was an *APOE*-dependent effect on this variant, we tested the association of p.E318G with CSF Aβ42 levels by stratifying it in the presence (+) or absence (−) of the *APOE* ε4 allele. We found that the risk of having Aβ deposition is greater for carriers of *PSEN1*, p.E318G and *APOE* ε4 together (OR = 18.3 CI = 2.0–166.8, p = 3.5×10^−3^) than those carrying *APOE* ε4 allele alone (OR = 4.5, CI = 3.4–6.0, p<1.0×10^−5^) (Table 4). These individuals are more likely to have a CSF biomarker profile similar consistent with AD (low CSF Aβ42, and high tau or ptau levels) (Figure 2A). p.E318G carriers who also carry *APOE* ε4+ allele (n = 20) exhibited significantly higher CSF tau (p = 0.04) and ptau (p = 0.01) levels and significantly lower CSF levels of Aβ42 (p = 0.02) compared to those that are p.E318G carriers but do not carry the *APOE* ε4 allele (Figure 2 A, B). We also found a significant interaction (p = 0.03) between *APOE* ε4+ and p.E318G in individuals with increased burden of Aβ deposition. Taken together, the results of the biomarker analyses suggest that *PSEN1*, p.E318G is associated with higher levels of neuronal loss (reflected by CSF tau and ptau levels) and with Aβ deposition (low Aβ42 CSF levels) in an *APOE* ε4-dependent fashion.

**Table 4:** Effect of the interaction of *PSEN1* p.E318G with *APOE* in individuals with and without Aβ deposition.

CSF samples		Lack of Aβ deposition	Aβ deposition	p value	OR (95%CI)
**E318G −**	**Apoe ε4 (+)**	105	299	1.7×10^−27^	**4.5 (3.4–6.0)**
	**Apoe ε4 (−)**	322	201		
**E318G +**	**Apoe ε4 (+)**	1	15	3.5×10^−3^	**18.3 (2.0–166.8)**
	**Apoe ε4 (−)**	11	9		

Samples were stratified based on the CSF Aβ42 levels as an approximation to the Aβ deposition. For ADNI-CSF series the cut-off was Aβ42 = 192 pg/mL and in WU-ADRC-CSF series we used a CSF Aβ42 = 500 pg/mL.

### Replication of the *PSEN1*, p.E318G-APOE interaction in large case-control datasets

Because the purpose of this endophenotype-based approach is to identify variants implicated in disease, we tested whether the *PSEN1*, p.E318G is associated with AD risk, tau/Aβ pathology or rate of cognitive decline in an APOE dependent manner.

Analyses of the association between *PSEN1* p.E318G and clinical AD status in an independent AD case-control series (n = 1,855, WU series) revealed that the risk of AD is significantly higher for p.E318G/*APOE* ε4 carriers (OR = 9.9 CI = 2.6–37.5, p = 1.7×10^−4^) compared to individuals carrying APOE ε4 alone (OR = 5.1, CI = 4.1–6.3, p = 3.2×10^−59^) (Table 5). This finding was replicated in an independent sample from the GERAD consortium (n = 4,058). In this dataset, the association of p.E318G with AD case-control status in the presence of at least one *APOE* ε4 allele (OR = 10.3, 95% CI = 4.1–25.5, p = 4.1×10^−8^) was double the risk for AD in the presence of *APOE* ε4 alone (OR = 4.1, 95% CI = 3.5–4.8, p = 1.1×10^−79^). In the joint-analysis of these two independent series (5,161 individuals), the risk of developing AD in the p.E318G/*APOE* ε4 carriers (OR = 10.1, 95% CI = 4.8–20.9, p = 9.0×10^−12^) is two-fold the AD risk of those that carry *APOE* ε4 allele alone (OR = 4.4, 95% CI = 3.9–5.0, p = 6.8×10^−139^) (Table 5).

**Table 5:** Replication analysis of *PSEN1* p.E318G interaction with *APOE* in AD case-control status.

Study	Strata		Cases	Controls	*P* value	OR (95% CI)
**WU**	**E318G +**	**Apoe ε4 (+)**	24	3	1.7×10^−4^	9.9 (2.6–37.5)
		**Apoe ε4 (−)**	21	26		
	**E318G −**	**Apoe ε4 (+)**	605	187	3.2×10^−59^	5.1 (4.1–6.3)
		**Apoe ε4 (−)**	381	608		
**GERAD**	**E318G +**	**Apoe ε4 (+)**	60	8	4.1×10^−8^	10.3 (4.1–25.5)
		**Apoe ε4 (−)**	24	33		
	**E318G −**	**Apoe ε4 (+)**	1660	282	1.1×10^−79^	4.1 (3.5–4.8)
		**Apoe ε4 (−)**	1169	818		
**WU+GERAD**	**E318G +**	**Apoe ε4 (+)**	84	11	9.0×10^−12^	10.1 (4.8–20.9)
		**Apoe ε4 (−)**	45	59		
	**E318G −**	**Apoe ε4 (+)**	2265	469	6.8×10^−139^	4.4 (3.9–5.0)
		**Apoe ε4 (−)**	1550	1426		

In fact, we found that individuals who are *APOE* ε4 heterozygous and also carry the p.E318G variant are at similar AD risk (OR = 10.7, 95% CI = 4.7–24.6, p = 2.5×10^−10^) as *APOE* ε4 homozygous (OR = 9.9, 95% CI = 7.2.9–13.6, p = 5.5×10^−76^) and are at double the AD risk compared to *APOE* ε4 heterozygous that are not carrying p.E318G (OR = 3.9, 95% CI = 3.4–4.4, p = 2.8×10^−106^) (Table 6, Figure 2C).

**Table 6:** *PSEN1*, p.E318G modifies Alzheimer's risk in *APOE* e4 carriers.

Study	Strata		Cases	Controls	*P* value	OR (95% CI)
**WU+GERAD**	**E318G +**	**Apoe ε4 (−)**	45	59	Ref	
		**Apoe ε4 (+)**	69	8	2.5×10^−10^	**10.7 (4.7–24.6)**
		**Apoe ε44**	15	2	1.0×10^−3^	9.3 (2.0–42.9)
	**E318G −**	**Apoe ε4 (−)**	1550	1426	Ref	
		**Apoe ε4 (+)**	1800	426	2.8×10^−106^	**3.9 (3.4–4.4)**
		**Apoe ε44**	465	43	3.4×10–74	9.9 (7.2–13.7)

In an independent analysis leveraging two prospective cohorts, the Religious Orders Study and Rush Memory and Aging Project, we confirmed a significant interaction between *APOE4* and p.E318G with burden of neuritic plaques at autopsy (n = 748; *P* = 0.01) but we failed to detect any significant association with neurofibrillary tangles (p = 0.47). Interestingly, the effect of *APOE* ε4 allele alone on neuritic plaques (n = 748, p = 4.5×10^−24^, Beta = 0.39) was increased by two fold the presence of p.E318G (n = 204, p = 0.08, Beta = 0.74). p.E318G has previously associated with lower cognitive performance [45]. We tested whether the interaction between *APOE4* and p.E318G affect the episodic memory. We found that there is trend between interaction between *APOE4* and p.E318G with episodic memory decline (p = 0.08).Furthermore, the significant effect of *APOE* ε4 allele on episodic memory decline (p = 1.7×10^−16^, Beta = −0.06) was modified by the presence of p.E318G (p = 0.14, Beta = −0.16).However, these interactions showed the predicted direction of effects for these phenotypes based on the results of the biomarker data: In the presence of at least one *APOE*-ε4 allele, p.E318G is associated with more Aβ plaques, faster cognitive decline and higher risk for AD.

### Family based and segregation analysis

The p.E318G variant has been associated with familial AD in different populations [42], [44], [46]. However, this association has not been consistently replicated [43], [47], [48], [49]. Our previous analyses indicate that in sporadic AD cases the effect of the p.E318G variant can be detected only in presence of the *APOE* ε4 allele. We wanted to analyze whether the same effect is found in familial cases. We genotyped probands from 565 total LOAD families and found the presence of *PSEN1* p.E318G in 30 families (MAF = 2.5%). *PSEN1* p.E318G exhibited a higher frequency in individuals with familial LOAD than those with sporadic LOAD (MAF = 1.6%, n = 3,989, p = 0.02) and a group of age matched control subjects (MAF = 1.5%, n = 830, p = 0.03). Next, we tested whether the association with familial LOAD was due to the interaction of p.E318G with *APOE*-ε4 allele. The presence of *APOE*-ε4 allele in p.E318G carriers in familial AD (70%, 21/30) was higher than that in sporadic AD (65%, 84/129) but not statistically significant (p = 0.61). On the other hand, *APOE*-ε4/p.E318G carriers in familial AD were significantly higher (p = 4.0×10^−4^) than those in the control group (15%, 10/69). Therefore, the risk conferred by *APOE*-ε4 and p.E318G carriers in familial AD (OR = 16.4, 95% CI = 5.6–48.2, p = 5.8×10^−8^) compared to the control group was higher than the risk associated with sporadic AD (OR = 10.1, 95% CI = 4.8–20.9, p = 9.0×10^−12^). These results suggest that higher risk of the p.E318G variant in familial cases is mostly due to the high frequency of *APOE* ε4 allele in this population [28].

Interestingly, the p.E318G variant has been reported in multigenerational families with AD [42], [50]. However, *PSEN1* p.E318G is not considered pathogenic in part due to the absence of conclusive evidence for cosegregation with AD [34], [43], [47], [48]. We observed 8 families (with more than two affected individuals carrying p.E318G) in which p.E318G segregates with disease (Figure 2D), even in the absence of *APOE*-ε4 allele (two families) (Figure S2). These families do not carry any other mutations in *APP, PSEN1, PSEN2*, *GRN* and *MAPT* genes [28]. In three additional families the cosegregation p.E318G with AD was inconclusive because only a few family members had been sampled and/or because p.E318G carriers were below the mean age of onset for AD in their respective families. Thus, using the largest sample of familial LOAD screened to date for the role of p.E318G in AD, we have demonstrated that minor allele p.E318G increases the risk of familial LOAD. Furthermore, p.E318G cosegregates with AD in 26% of all the familial LOAD carriers.

### Effect on age at onset of AD

Carriers of *PSEN1*, p.E318G have been reported across a wide range of ages (45 to 93 yrs.) [42], [44], [46], [50]. Thus, we tested whether *PSEN1*, p.E318G affects AAO regardless of the APOE genotype; we found that *PSEN1*, p.E318G carriers have a lower AAO than non-carriers (73.9 yr. vs. 78.2 yr.; p = 0.01) (Figure S3).

## Discussion

Resequencing genes in individuals from the extremes of the biomarker distribution constitutes a powerful and efficient strategy to identify functional sequence variants associated with complex traits [10]. CSF-based biomarker profiles have proven to be powerful tools in endophenotype-oriented approaches, by which we have been able to identify common genetic variants associated with the rate of progression, AAO or the risk of AD [11], [14], [17], [18], [51]. Previously, we identified a pathogenic mutation in a family with LOAD within the *PSEN1* gene by selecting the top and bottom 5% from the distributions of CSF levels of Aβ40, Aβ42, and Aβ42/40 ratio [29]. Here, we have used a novel and powerful approach by using next-generation sequencing to sequence individuals with extreme phenotypes: individuals from the bottom and top 15% of Aβ42, tau, or ptau CSF levels.

### Pathogenic mutations and high-risk AD variants

Previous data have suggested that mutations in *APP*, *PSEN1*, and *PSEN2* genes only cause early-onset familial AD. However, this study and previous studies from our group [28], [52] indicate that pathogenic mutations in these genes can be also found in late-onset familial and sporadic AD cases. In this study, we observed a known and confirmed pathogenic mutation (*PSEN1* p.A426P, rs63751223) in one individual (57 years old) without a clear family history of dementia, out of 258 individuals (CDR>0), which constitutes 0.3% of AD cases.

In a previous study, Cruchaga et al, found that 2.3% of families with multiple members affected by LOAD carried pathogenic mutations [28]. In this study, we expanded our analyses to sporadic cases, which constitute 95% of the total number of AD cases. Although we found only one case with a pathogenic mutation (0.3%), this could be an underestimate because both of the novel mutations, *PSEN2*: p.G270S and *MAPT* p.T263P were found in single cases that met biomarker criteria for AD. A novel variant in *GRN*, p.C247Y and a known variant in *PSEN1*, p.R35Q were found in demented individuals with a non-AD CSF profile suggesting another type of dementia. However, without segregation analyses, additional functional studies are required to determine the potential pathogenicity of these variants.

The classification of mutations as not pathogenic, possibly pathogenic, probably pathogenic and definitely pathogenic based on segregation analyses, amino acid conservation, effects on Aβ metabolism in *in vitro* studies, association studies and presence in healthy individuals has been useful in prioritizing mutations and their likelihood of affecting risk for disease [47]. However, this classification is likely to miss variants with a smaller but real effect (OR>2.0) on risk for sporadic AD. The variant *GRN*, p.P458L is classified as non-pathogenic [34] due to fact that it was reported in an ALS/FTD patient and in 25 out of 492 controls (MAF = 2.5%) [53]. However, this variant is not reported in the EVS server (6,515 exomes) (EVS-v.0.0.18, (February 8, 2013) or in our control population of 824 samples (Table 2). Here, this variant was found in an individual with early onset dementia and with typical biomarker criteria for AD. *PSEN2*, p.R71W has been classified as non-pathogenic because it was reported in controls and EOAD cases [34]. However, in a previous study the frequency of the p.R71W variant in AD cases was significantly higher than in controls (n = 3,152, p = 9.0×10^−4^ OR = 6.45; 95%CI = 1.95–21.39) and carriers have a significantly earlier age at onset than affected non-carriers (p.R71W: 70.2 vs. 76.7, p = 5.0×10^−4^), suggesting that this variant could be a modifier of LOAD risk [28]. Here, we found the same trend, *PSEN2* p.R71W was also found to be present more frequently in clinical cases than in controls (p = 0.03, OR = 10.3, 95%CI = 1.1–96.2). However, it did not reach statistical significance in individuals with Aβ deposition (p = 0.27, OR = 3.4, 95%CI = 0.38–30.7).

### *PSEN1* p.E318G increases the risk of AD in *APOE* ε4 allele carriers

The *PSEN1*, p.E318G variant has been considered to be a non-pathogenic variant, because it has been found in non-demented individuals [43], [48], [49] and the absence of conclusive evidence for cosegregation with AD [43]. However, it has been suggested that phenocopies, potential presymptomatic individuals, reduced penetrance and gene by gene interactions complicate the interpretation of the p.E318G variant in familial and sporadic LOAD [42], [44]. This is the first study to systematically screen the presence of *PSEN1* p.E318G in a large (n = 565) clinical series of well-characterized families densely affected by LOAD with no mutations in *APP, PSEN2, GRN* or *MAPT* genes. *PSEN1* p.E318G was found in 5.3% and cosegregated with the disease in 1.4% of all families. We also found that *PSEN1* p.E318G exhibited a higher frequency in familial LOAD than in sporadic LOAD (p = 0.025), supporting earlier findings that the p.E318G variant has higher frequencies among AD cases with a family history of AD in different populations [42], [44], [46]. Additionally, our analyses indicate that *PSEN1* p.E318G carriers have an average age at onset that is 4.3 years earlier than that in non-carriers (73.9 yr. vs. 78.2 yr). Putative pathogenic variants in genes that cause late-onset rather than early-onset dementia could have a less severe effect on protein function due to genetic or environmental modifiers [28]. Our CSF biomarker analyses suggested that *PSEN1* p.E318G was associated with higher levels of neuronal loss (reflected by high CSF tau and ptau levels) and with Aβ deposition (low Aβ42 CSF levels) in an *APOE* ε4-dependent fashion. Furthermore, in the largest AD case-control series (n = 5,161) analyzed for the interaction between *PSEN1* p.E318G and *APOE* ε4 allele to date, we found that the presence of p.E318G and APOE ε4 doubles the risk for AD (OR = 10.3, 95% CI = 4.1–25.5) compared to the risk with the presence of *APOE* ε4 alone (OR = 4.1, 95% CI = 3.5–4.8). There are several reports of variants that modify the risk of AD in *APOE* ε4 carriers such as α-1-antichymotrypsin (ACT) gene (*APOE* ε4/ACT, [OR = 6.4, non 95% CI reported]) [54], Cholesteryl ester transfer protein (CETP) gene (*APOE* ε4/CETP [–629] C allele [OR 7.12, non 95% CI reported]) [55], GRB-associated binding protein 2 (GAB2) gene (*APOE* ε4/rs2373115 genotype GG [OR = 2.36, 95% CI 1.55–3.58]) [56], CUG triplet repeat, and RNA binding protein 2 (CUGBP2) gene (*APOE ε4/ε4*/rs62209 [OR = 1.75, 95% CI 1.27–2.41]) [57]. However, all these variants have a modest effect increasing the risk due to *APOE* ε4 allele. Here, we provided evidence of a low frequency variant in *PSEN1* gene with a significant effect on the AD risk in *APOE* ε4 carriers (OR = 10.7, 95% CI = 4.7–24.6) comparable only to the effect of a second *APOE* ε4 allele (OR = 9.9, 95% CI = 7.2.9–13.6). Moreover, we also found that in the presence of at least one *APOE* ε4 allele, p.E318G is associated with more Aβ plaques and faster cognitive decline, as recently reported for a low frequency variant in complement receptor 1 (CR1) [58] In addition, p.E318G has previously associated with lower cognitive performance, which support our findings of cognitive decline [45]. The interaction of the p.E318G with *APOE* ε4 allele was replicated in four different datasets: the CSF dataset (discovery set), WU_ADRC case-control dataset, GERAD1 and the Religious Orders Study and Rush Memory and Aging Project, indicating that this association and interaction is not a type I error, but a real association. All these results together support the role of *PSEN1* p.E318G as one of the most important modifiers of the risk of LOAD reported to date.

Functional studies, especially concerning the effect on Aβ metabolism *in vitro*, have further questioned the pathogenicity of the p.E318G variant. One study showed no alteration in the production of Aβ42 induced by p.E318G [43]. However, a recent study using skin fibroblasts from individuals with the p.E318G variation showed an increase in the production of Aβ40, a decrease in Aβ42 and a subsequent significant reduction in the Aβ42/Aβ40 ratio compare to non-carriers [42], along with a lack of an inhibitory effect of the exon 9 loop in the presence of the p.E318G variant reported by an independent study [59]. It has been proposed that the activation of γ-secretase results from a cleavage-induced conformational change that relieves the inhibitory effect of the intact exon 9 loop, which is mediated by occupying the substrate-binding site on the immature enzyme before it is cleaved [59]. It was reported that p.E318G abolishes the inhibitory effect of the intact exon 9 loop, which favors the production of Aβ40 [59]. It was also reported that p.E318G affects the processing of *PSEN1* by reducing the amount of N-terminal fragment that is generated after cleavage [60], and augments levels of neuronal cell death after overexpression [61]. We suggest that another approach to test the impact of pathogenic mutations on Aβ metabolism is to examine the effect on the CSF biomarker levels. Most of the published data about CSF biomarkers reveal that *PSEN1* gene mutation carriers display a typical AD biomarker signature with low CSF levels of Aβ42 and high CSF tau levels [13], [20]. There is no published data on the levels of CSF biomarkers for *PSEN1*, p.E318G carriers. Here, for the first time we demonstrate that *PSEN1*, p.E318G/*APOE* ε4 carriers have a CSF biomarker profile similar to AD cases.

In summary, these results highlight the relative enrichment of low frequency variants in six genes involved in AD and FTD that are at the extremes of the distribution of CSF biomarker levels [29]. We provide evidence that the *PSEN1*, p.E318G variant increases the risk for AD in *APOE* ε4 heterozygous, equivalent to that of *APOE* ε4 homozygous. We also found that p.E318G increases the risk of familial LOAD and cosegregates with AD in 26% of all the familial LOAD carriers. All these findings have important implications for genetic counseling since *PSEN1*, p.E318G is currently considered a non-pathogenic variant [50].

By using CSF biomarker levels as a quantitative trait, we were able to identify a low frequency variant associated with AD risk, *PSEN1*, p.E318G. This association is mediated by a SNP-by-SNP interaction, which has not been found using the standard case-control design [43], [48], [49]. Together, these results indicate that there are potentially many more low frequency variants associated with complex disease, and that the association results from complex interactions. We were able to identify the association of *PSEN1*, p.E318G with risk for AD and its interaction with the *APOE* ε4 allele because both genes are known to be associated with AD. However, the identification of such an association and interactions in a genome-wide approach remains still challenging and requires novel, powerful approaches.

We believe that this endophenotype-based approach is a good alternative to case-control studies and can allow us to gain a better understanding of both the genetic architecture and pathophysiology of LOAD [17], [18]. In terms of genetics and factors that may explain some of the missing hereditability of complex diseases, these results are important because they are a clear example of low frequency variants that are associated with disease and how such associations are due to epistatic gene by gene interactions.

## Materials and Methods

### Ethics statement

The Institutional Review Board (IRB) at the Washington University School of Medicine in Saint Louis approved the study. Prior to their participation, a written informed consent was reviewed and obtained from family members. The Human Research Protection Office (HRPO) approval number for our ADRC Genetics Core family studies is 93-0006.

### Samples

Two CSF series were used for this study. A total sample of 475 individuals enrolled in longitudinal studies at the Alzheimer's disease Research Center at Washington University School of Medicine (ADRC) and 259 participants of the Alzheimer's disease Neuroimaging Initiative (ADNI) were used in this study. A subset of 145 participants from ADRC and 67 from ADNI were included in the discovery series (two DNA pools). CSF samples were from individuals of European descent. In the WU-ADRC-CSF series: 60% of sample is female, ranging from 37–91 years of age. 73% of the sample has a clinical dementia rating (CDR) of 0 (cognitively normal) and 39% of the individuals carry at least one APOE ε4 allele. In the ADNI-CSF series: 44% of sample is female, ranging from 56–91 years of age. 60% of the sample has a CDR higher than 0 (demented) and 47% are APOE ε4 allele positive. Table 1 summarizes the demographic data for the CSF series. Covariate-adjusted residuals of CSF Aβ42, tau and p-tau were used to define the pools (see statistical analysis, Table S3 in Text S1). 114 individuals in the bottom 15% of CSF Aβ42 levels or individuals in the top 15% of CSF tau or p-tau levels were included in a pool. The second pool consisted of 98 individuals in the top 15% of CSF Aβ42 or individuals in the bottom 15% of tau and p-tau181 levels (Table 1).

The Religious Orders Study (ROS) and the Rush Memory and Aging Project (MAP) recruit participants without known dementia who agree to annual clinical evaluations and sign an Anatomic Gift Act donating their brains at death. The full cohort with genotype data included 1,708 subjects (817 ROS and 891 MAP). The mean age at enrollment was 78.5 years and 69.1% were female. At the last evaluation, 24.9% met clinical diagnostic criteria for AD and 21.8% had mild cognitive impairment. The summary measure of global cognitive performance was based on annual assessments of 17 neuropsychiatric tests. A nested autopsy cohort consisted of 651 deceased subjects (376 ROS and 275 MAP); mean age at death was 81.5 years and 37.6% were male. Proximate to death, 40.9% of subjects included in the autopsy cohort met clinical diagnostic criteria for AD. Bielschowsky silver stain was used to visualize neurofibrillary tangles in tissue sections from the midfrontal, middle temporal, inferior parietal, and entorhinal cortices, and the hippocampal CA1 sector. A quantitative composite score for neurofibrillary tangle pathologic burden was created by dividing the raw counts in each region by the standard deviation of the region specific counts, and then averaging the scaled counts over the 5 brain regions to create a single standardized summary measure. Additional details of the ROS and MAP cohorts as well as the cognitive and pathologic phenotypes are described in prior publications [58], [62]. Follow-up series included 1,031 sporadic AD cases, 824 unrelated elderly cognitively normal controls and a single case from NIA-LOAD families (n = 595) [28]. All these samples are independent of the CSF samples. Cases received a diagnosis of dementia of the Alzheimer's type (DAT), using criteria equivalent to the National Institute of Neurological and Communication Disorders and Stroke-Alzheimer's Disease and Related Disorders Association for probable AD [63], [64]. Controls received the same assessment as the cases but were non-demented. All individuals were of European descent and written consent was obtained from all participants.

DNA from ROS and MAP subjects was extracted from whole blood, lymphocytes or frozen post-mortem brain tissue and genotyped on the Affymetrix Genechip 6.0 platform, as previously described [58]. Following standard QC procedures, imputation was performed using MACH software (version 1.0.16a) and HapMap release 22 CEU (build 36) as a reference.

### Statistical and association analyses

Association of Aβ42, tau and p-tau181 with genetic variants was analyzed as previously reported [14], [17], [18]. Briefly, Aβ42, tau and p-tau181 values were log transformed to approximate a normal distribution. Because the CSF biomarker levels were measured using different platforms (Innotest plate ELISA vs. AlzBia3 bead-based ELISA, respectively) we were not able to combine the raw data. For the combined analyses we standardized the mean of the log transformed values from each dataset to zero. A stepwise discriminant analysis identified CDR, *APOE* genotype, gender and age as significant covariates in both series (Table S1b in Text S1) [17], [18]. No significant differences in the transformed and standardized CSF values for different series were found (Table S1b in Text S1).

CSF biomarker levels were used as a quantitative trait for most analyses. It has been shown that CSF Aβ42 is an accurate predictor of brain amyloid burden regardless of clinical diagnosis [39]. Therefore, the Aβ plaque deposition was assumed using the biomarker levels as a dichotomous variable (low and high CSF Aβ42). Levels of CSF biomarkers were as follows: for the ADNI-CSF series the cut-off was Aβ42<192 pg/mL [35]. In the WU-ADRC-CSF series, we used CSF Aβ42<500 pg/mL as the cut-off [39].

We used Plink (http://pngu.mgh.harvard.edu/~purcell/plink/) to analyze the association of variants (individually or collapsed by gene) with CSF biomarker levels. Age, gender and site were included as covariates. In order to determine whether the association of variants with CSF biomarker levels was driven by case-control status we included clinical dementia rating (CDR) or CSF Aβ42 levels as a covariate in the model or stratified the data by case control status. We also performed analyses including APOE genotype as a covariate. Association with AAO was carried out using the Kaplan-Meier method and tested for significant differences, using a log-rank test [17].

Fisher's exact test was used to compare the frequency of each variant and collapse by gene in the case control series defined by CDR or CSF Aβ42 levels (Table 3). All variants were included in the model independent of their pathogenicity.

Analyses of SNP effects on global cognitive decline in ROS and MAP were performed as in prior publications [62]. Briefly, we first fit linear mixed effects models using the global cognitive summary measure in order to characterize individual paths of change, adjusted for age, sex, years of education, and their interactions with time. At least two longitudinal measures of cognition were required for inclusion in these analyses, for which data on 1,593 subjects was available. We then used these person-specific, residual cognitive decline slopes as the outcome variable in our linear regression models, with each SNP of interest coded additively relative to the minor allele, and further adjusted for study membership (ROS vs. MAP) and the first 3 principal components from population structure analysis. For analyses of neurofibrillary tangle burden, linear regression was used to relate SNPs to the pathologic summary measure, adjusting for age at death, study membership, and 3 principal components. Because the data were skewed, square root of the scaled neurofibrillary tangle burden summary score was used in analyses.

### Pooled sequencing analysis

Pooled-DNA sequencing was performed, as previously described by Druley TE et al. [28], [52], [65], [66]. Briefly, equimolar amounts of individual DNA samples were pooled together after being measured using Quant-iT PicoGreen reagent. Two different pools with 100 ng of DNA from 114 and 98 individuals were made. The coding exons and flanking regions (a minimum of 50 bp each side) were individually PCR amplified using specific primers and Pfu Ultra high-fidelity polymerase (Stratagene). An average of 20 diploid genomes (approximately 0.14 ng DNA) per individual were used as input into a total of 62 PCR reactions that covered 46,319 bases from the 6 genes. PCR products were cleaned using QIAquick PCR purification kits, quantified using Quant-iT PicoGreen reagent and ligated in equimolar amounts using T4 Ligase and T4 Polynucleotide Kinase. After ligation, concatenated PCR products were randomly sheared by sonication and prepared for sequencing on an Illumina Genome Analyzer IIx (GAIIx) according to the manufacturer's specifications. pCMV6-XL5 amplicon (1908 base pairs) was included in the reaction as a negative control. As positive controls, ten different constructs (p53 gene) with synthetically engineered mutations at a relative frequency of one mutated copy per 250 normal copies was amplified and pooled with the pcr products. Six DNA samples heterozygous for previously known mutants in *GRN, PSEN1, MAPT* genes were also included.

Single reads (36 bp) were aligned to the human genome reference assembly build 36.1 (hg18) using SPLINTER [67]. SPLINTER uses the positive control to estimate sensitivity and specificity for variant calling. The wild type: mutant ratio in the positive control is similar to the relative frequency expected for a single mutation in one pool (1 chromosome mutated in 125 samples = 1/250). SPLINTER uses the negative control (first 900 bp) to model the errors across the 36-bp Illumina reads and to create an error model from each sequencing run of the machine. Based on the error model SPLINTER calculates a p-value for the probability that a predicted variant is a true positive. A p-value at which all mutants in the positive controls were identified was defined as the cut-off value for the best sensitivity and specificity. All mutants included as part of the amplified positive control vector were found upon achieving >30-fold coverage at mutated sites (sensitivity = 100%) and only ∼80 sites in the 1908 bp negative control vector were predicted to be polymorphic (specificity = ∼95%). The variants with a p-value below this cut-off value were considered for follow-up confirmation. All rare missense or splice site variants (with an estimated allelic frequency less than 5%) were then validated by Sequenom and KASPar genotyping in each individual included in the pools [28], [52], [66]. The validated SNPs were then genotyped in all members of the WU-ADRC-CSF and ADNI-CSF series. Common variants (>5%) and synonymous variants were not followed up.

An average coverage of 30X-fold per allele per pool is the minimum coverage necessary to get an optimal positive predictive value for the SNP-calling algorithm [67]. The necessary number of lanes to obtain a minimum of 30-fold coverage per base and sample were run (Table S2 in Text S1).

The WU-ADRC samples were genotyped with the Illumina 610 or OmniExpress. The ADNI samples were genotyped with the Illumina 610 chip. Prior to association analysis, all samples and genotypes underwent stringent quality control (QC). Genotype data were cleaned applying a minimum call rate for SNPs and individuals (98%) and minimum minor allele frequencies (0.02). SNPs not in Hardy-Weinberg equilibrium (P<1×10^−6^) were excluded. The QC cleaning steps were applied for each genotyping array separately. We tested for unanticipated duplicates and cryptic relatedness using pairwise genome-wide estimates of proportion identity-by-descent. When a pair of identical samples or a pair of samples with cryptic relatedness was identified, the sample from the WU-ADRC or samples with a higher number of SNPs passing QC were prioritized. Eigenstrat was used to calculate principal component factors for each sample and confirm the ethnicity of the samples [68]. The 1000 Genome Project data (June 2011 release) and Beagle software were used to impute up to 6 million SNPs. SNPs with a Beagle R2 of 0.3 or lower, a minor allele frequency (MAF) lower than 0.05, out of Hardy-Weinberg equilibrium (p<1×10-6), a call rate lower than 95% or a Gprobs score lower than 0.90 were removed. A total of 5,815,690 SNPs passed the QC process.

We used PLINK to select the list of SNPs in the gene region (approximately 250 kb of flanking sequence each side) from the imputed data. These SNPs were pruned with an r^2^ cutoff of 0.8.. The simple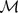 method [38] was used to calculate the number of informative SNPs within the genomic region for each gene. This measure was then used in a Bonferroni adjustment to estimate the significance threshold. Significant SNPs that were imputed or have a MAF<10% were directly genotyped in all the samples to confirm the association.

### Bioinformatics

The AD&FTD mutation database [34] was used to identify sequence variants previously found in other studies of early onset familial dementia and to determine whether or not they are considered to be disease-causative variants. The sequencing data from the 1,000 Genome Project and the Exome Variant Server data base (http://evs.gs.washington.edu/EVS/) were used to estimate the frequency of novel and rare (minor allele frequency less than 5%) missense, nonsense and splice site variants in samples unselected for studies of AD. Conservation was determined by using the GERP score, which calculates the conservation of each nucleotide in multi-species alignment. A site was called conserved when the GERP score was greater than or equal to 4 [69], [70].

### ADNI material and methods

Data used in the preparation of this article were obtained from the ADNI database (www.loni.ucla.edu\ADNI). The ADNI was launched in 2003 by the National Institute on Aging, the National Institute of Biomedical Imaging and Bioengineering, the Food and Drug Administration, private pharmaceutical companies and non-profit organizations, as a $60 million, 5-year public-private partnership. The Principal Investigator of this initiative is Michael W. Weiner, M.D. ADNI is the result of efforts of many co-investigators from a broad range of academic institutions and private corporations, and subjects have been recruited from over 50 sites across the U.S. and Canada. The initial goal of ADNI was to recruit 800 adults, ages 55 to 90, to participate in the research -approximately 200 cognitively normal older individuals to be followed for 3 years, 400 people with MCI to be followed for 3 years, and 200 people with early AD to be followed for 2 years.” For up-to-date information see www.adni-info.org.

### GERAD data information

Data used in the preparation of this article were obtained from the Genetic and Environmental Risk for Alzheimer's disease (GERAD1) Consortium [71]. The GERAD1 sample comprised up to 3941 AD cases and 7848 controls. A subset of this sample has been used in this study, comprising 3333 cases and 1225 elderly screened controls genotyped at the Sanger Institute on the Illumina 610-quad chip. These samples were recruited by the Medical Research Council (MRC) Genetic Resource for AD (Cardiff University; Kings College London; Cambridge University; Trinity College Dublin), the Alzheimer's Research Trust (ART) Collaboration (University of Nottingham; University of Manchester; University of Southampton; University of Bristol; Queen's University Belfast; the Oxford Project to Investigate Memory and Ageing (OPTIMA), Oxford University); Washington University, St Louis, United States; MRC PRION Unit, University College London; London and the South East Region AD project (LASER-AD), University College London; Competence Network of Dementia (CND) and Department of Psychiatry, University of Bonn, Germany and the National Institute of Mental Health (NIMH) AD Genetics Initiative. All AD cases met criteria for either probable (NINCDS-ADRDA, DSM-IV) or definite (CERAD) AD. All elderly controls were screened for dementia using the MMSE or ADAS-cog, were determined to be free from dementia at neuropathological examination or had a Braak score of 2.5 or lower.

## Supporting Information

Figure S1 Study design.
(TIF)

Figure S2 Pedigree a family with p.E318G carriers illustrating the segregation analysis and the absence of *APOE* ε4. A/G is the genotype for p.E318G variant and 3/3, is the APOE genotype. * Symbol means confirmed AD by autopsy.
(TIF)

Figure S3 Survival curves comparing age at onset of LOAD between the different genotypes of Psen1, p.E318G. Survival fractions were calculated using the Kaplan-Meier method and significant differences were calculated by Log-rank test. Association with age at onset was calculated in 21 families with at least two AD cases carrier.
(TIF)

Text S1 Information about the known variants.
(DOCX)
