# Supplementary figures and images for: The PSEN1, p.E318G Variant Increases the Risk of Alzheimer's Disease in APOE-ε4 Carriers
Source: PLoS Genet. 2013 Aug 22;9(8):e1003685. doi: 10.1371/journal.pgen.1003685 (PMC3750021; doi:10.1371/journal.pgen.1003685)

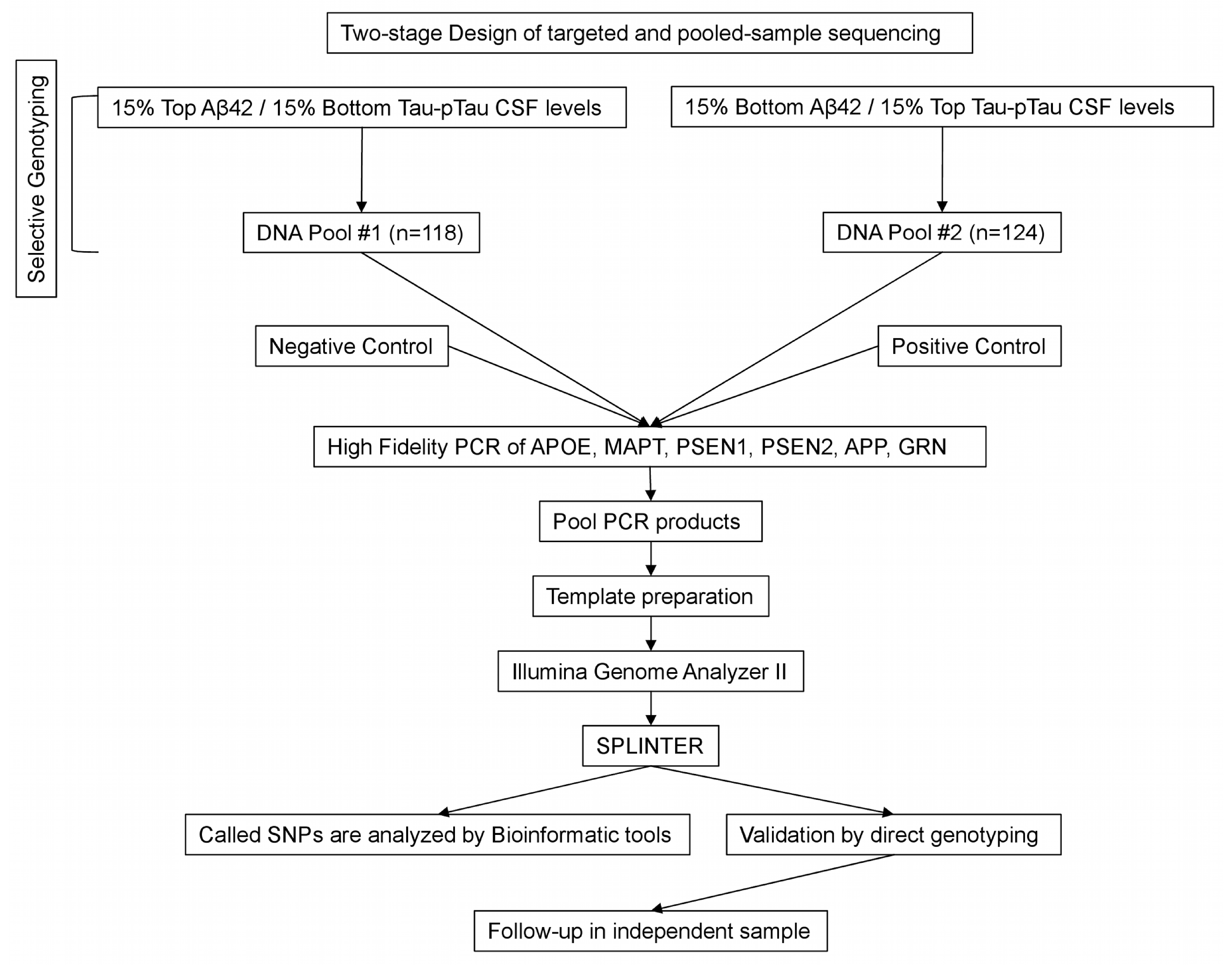

Supplement: Figure S1 — Study design. (TIF) [file pgen.1003685.s001.tif]

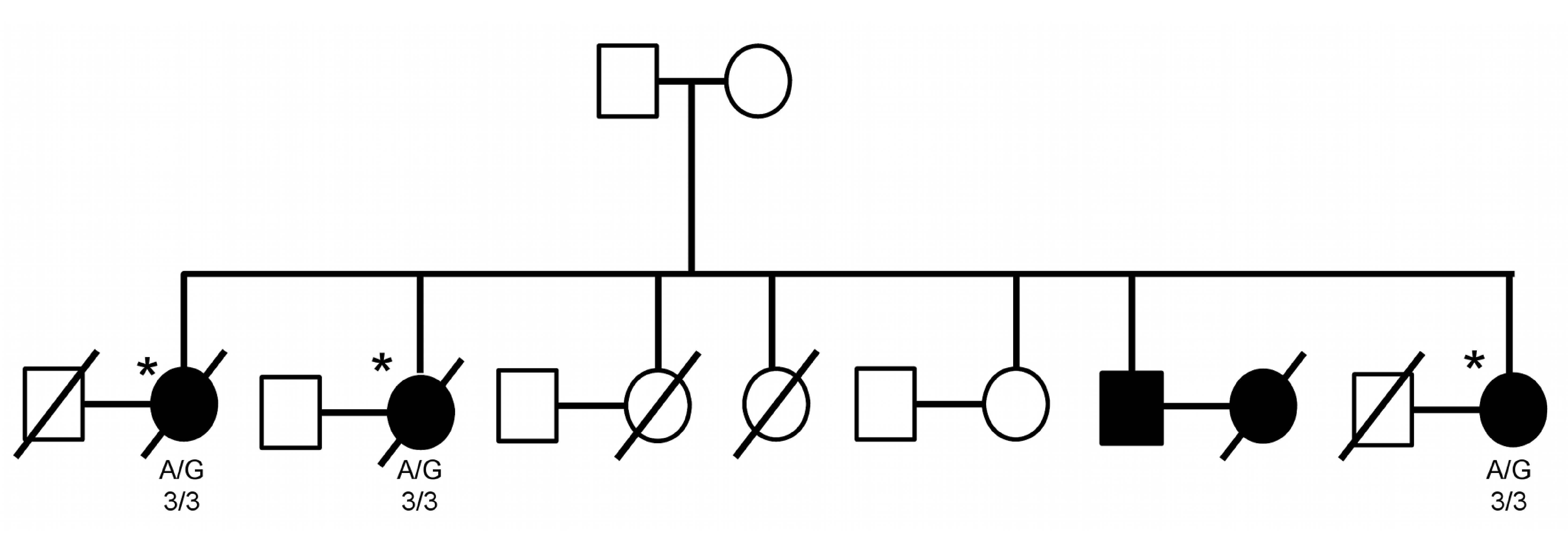

Supplement: Figure S2 — Pedigree a family with p.E318G carriers illustrating the segregation analysis and the absence of APOE ε4. A/G is the genotype for p.E318G variant and 3/3, is the APOE genotype. * Symbol means confirmed AD by autopsy. (TIF) [file pgen.1003685.s002.tif]

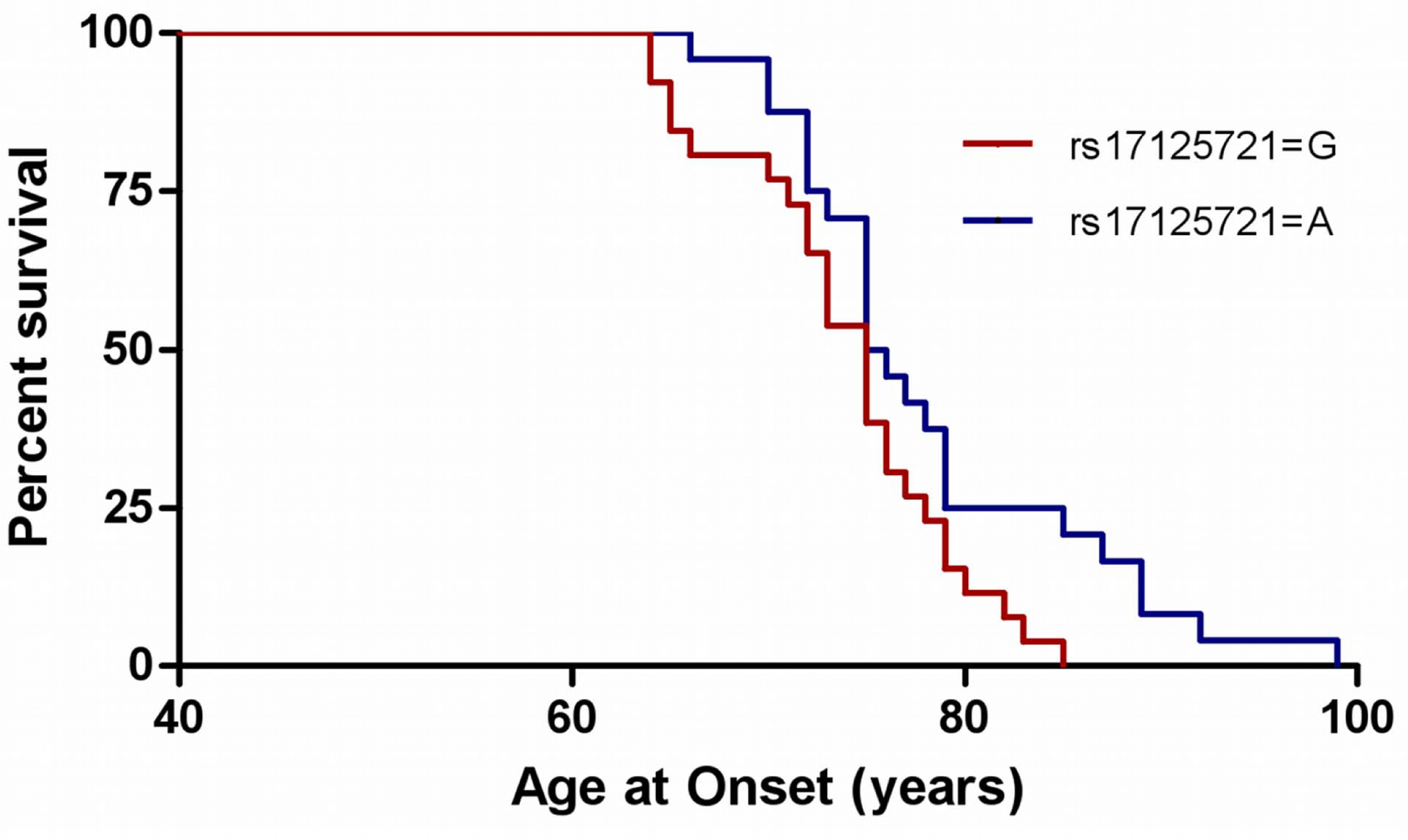

Supplement: Figure S3 — Survival curves comparing age at onset of LOAD between the different genotypes of Psen1, p.E318G. Survival fractions were calculated using the Kaplan-Meier method and significant differences were calculated by Log-rank test. Association with age at onset was calculated in 21 families with at least two AD cases carrier. (TIF) [file pgen.1003685.s003.tif]
