# Supplementary material for: The PSEN1, p.E318G Variant Increases the Risk of Alzheimer's Disease in APOE-ε4 Carriers
Source: PLoS Genet. 2013 Aug 22;9(8):e1003685. doi: 10.1371/journal.pgen.1003685 (PMC3750021; doi:10.1371/journal.pgen.1003685)
Supplement: Text S1 — Information about the known variants. (DOCX) [file pgen.1003685.s004.docx]

**The *PSEN1*, p.E318G variant increases the risk of Alzheimer's disease in *APOE*-ε4 carriers**

**Authors and Affiliations**

Benitez BA^1§^, Karch CM^1^, Cai Y^1^, Jin SC^1^, Cooper B^1^, Carrell D^1^, Bertelsen S^1^, Lori Chibnik^3,4,5^, Julie A. Schneider^6^, David A. Bennett^6^ Alzheimer’s Disease Neuroimaging Initiative (ADNI)^†^, Genetic and Environmental Risk for Alzheimer's Disease Consortium (GERAD1)^γ^, Fagan AM^6,8^, Hotlzman D^6,8^, Morris JC^6,8^, Goate AM^1,6,7,8^, Cruchaga C^1,8§^*,

1. Department of Psychiatry, Washington University, St. Louis, MO, USA. 2. Program in Translational NeuroPsychiatric Genomics, Institute for the Neurosciences Department of Neurology, Brigham and Women's Hospital, Boston, MA 02115, 3. Harvard Medical School, Boston, MA 02115, 4. Program in Medical and Population Genetics, Broad Institute of Harvard University and M.I.T., Cambridge, MA 02142. 5. Rush Alzheimer’s Disease Center and Department of Neurological Sciences, Rush University Medical Center, Chicago, IL 60062. 6. Department of Neurology, Washington University, St. Louis, MO, USA. 7. Department of Genetics, Washington University, St. Louis, MO, USA 8. Hope Center Program on Protein Aggregation and Neurodegeneration, Washington University St. Louis, MO, USA

^§^These authors contributed equally to this work.

^*^To whom correspondence should be addressed at: Department of Psychiatry, Washington University School of Medicine, 660 South Euclid Avenue B8134, St. Louis, MO 63110. E-mail: [cruchagac@psychiatry.wustl.edu](mailto:cruchagac@psychiatry.wustl.edu), tel. 314-286-0546, fax. 314-747-2983

^†^Data used in preparation of this article were obtained from the Alzheimer’s Disease Neuroimaging Initiative (ADNI) database (adni.loni.ucla.edu). As such, the investigators within the ADNI contributed to the design and implementation of ADNI and/or provided data but did not participate in analysis or writing of this report. A complete listing of ADNI investigators can be found at: <http://adni.loni.ucla.edu/wp-content/uploads/how_to_apply/ADNI_Acknowledgement_List.pdf>

^γ^ Data used in the preparation of this article were obtained from the Genetic and Environmental Risk for Alzheimer’s disease (GERAD1) Consortium. As such, the investigators within the GERAD1 consortia contributed to the design and implementation of GERAD1 and/or provided data but did not participate in analysis or writing of this report. A full list of GERAD1 investigators can be included in either supplementary content or acknowledgements.

**Genetic and Environmental Risk for Alzheimer’s disease (GERAD1) Consortium Author List**

Denise Harold^1^, Rebecca Sims^1^, Amy Gerrish^1^, Jade Chapman^1^, Valentina Moskvina^1^, Richard Abraham^1^, Paul Hollingworth^1^, Marian Hamshere^1^, Jaspreet Singh Pahwa^1^, Kimberley Dowzell^1^, Amy Williams^1^, Nicola Jones^1^, Charlene Thomas^1^, Alexandra Stretton^1^, Angharad Morgan^1^, Kate Williams^1^, Simon Lovestone^2^, John Powell^2^, Petroula Proitsi^2^, Michelle K Lupton^2^, Carol Brayne^3^, David C. Rubinsztein^4^, Michael Gill^5^, Brian Lawlor^5^, Aoibhinn Lynch^5^, Kevin Morgan^6^, Kristelle Brown^6^, Peter Passmore^7^, David Craig^7^, Bernadette McGuinness^7^, Janet A Johnston^7^, Stephen Todd^7^, Clive Holmes^8^, David Mann^9^, A. David Smith^10^, Seth Love^11^, Patrick G. Kehoe^11^, John Hardy^12^, Rita Guerreiro^13,33^, Andrew Singleton^13^, Simon Mead^14^, Nick Fox^15^, Martin Rossor^15^, John Collinge^14^, Wolfgang Maier^16^, Frank Jessen^16^, Reiner Heun^16^, Britta Schürmann^16,17^, Alfredo Ramirez^16^, Christine Herold^34^, André Lacour^34^, Dmitriy Drichel^34^, Hendrik van den Bussche^18^, Isabella Heuser^19^, Johannes Kornhuber^20^, Jens Wiltfang^21^, Martin Dichgans^22,23^, Lutz Frölich^24^, Harald Hampel^25^, Michael Hüll^26^, Dan Rujescu^27^, Alison Goate^28^, John S.K. Kauwe^29^, Carlos Cruchaga^28^, Petra Nowotny^28^, John C. Morris^28^, Kevin Mayo^28^, Gill Livingston^30^, Nicholas J. Bass^30^, Hugh Gurling^30^, Andrew McQuillin^30^, Rhian Gwilliam^31^, Panagiotis Deloukas^31^, Markus M. Nöthen^32^, Peter Holmans^1^, Michael O’Donovan^1^, Michael J.Owen^1^, Julie Williams^1^.

**Affiliations**

^1^ Medical Research Council (MRC) Centre for Neuropsychiatric Genetics and Genomics, Neurosciences and Mental Health Research Institute, Department of Psychological Medicine and Neurology, School of Medicine, Cardiff University, Cardiff, UK.

^2^ King's College London, Institute of Psychiatry, Department of Neuroscience, De Crespigny Park, Denmark Hill, London.

^3^ Institute of Public Health, University of Cambridge, Cambridge, UK.

^4^ Cambridge Institute for Medical Research, University of Cambridge, Cambridge, UK.

^5^ Mercer's Institute for Research on Aging, St. James Hospital and Trinity College, Dublin, Ireland.

^6^ Institute of Genetics, Queen's Medical Centre, University of Nottingham, UK.

^7^ Ageing Group, Centre for Public Health, School of Medicine, Dentistry and Biomedical Sciences, Queen's University Belfast, UK.

^8^ Division of Clinical Neurosciences, School of Medicine, University of Southampton, Southampton, UK.

^9^ Clinical Neuroscience Research Group, Greater Manchester Neurosciences Centre, University of Manchester, Salford, UK.

^10^ Oxford Project to Investigate Memory and Ageing (OPTIMA), University of Oxford, Level 4, John Radcliffe Hospital, Oxford, UK.

^11^ University of Bristol Institute of Clinical Neurosciences, School of Clinical Sciences, Frenchay Hospital, Bristol, UK

^12^ Department of Molecular Neuroscience and Reta Lilla Weston Laboratories, Institute of Neurology, UCL, London, UK.

^13^ Laboratory of Neurogenetics, National Institute on Aging, National Institutes of Health, Bethesda, Maryland, United States of America

^14^ MRC Prion Unit, Department of Neurodegenerative Disease, UCL Institute of Neurology, London, UK.

^15^ Dementia Research Centre, Department of Neurodegenerative Diseases, University College London, Institute of Neurology, London, UK.

^16^ Department of Psychiatry, University of Bonn, Sigmund-Freud-Straβe 25, 53105 Bonn, Germany.

^17^ Institute for Molecular Psychiatry, University of Bonn, Bonn, Germany

^18^ Institute of Primary Medical Care, University Medical Center Hamburg-Eppendorf, Germany.

^19^ Department of Psychiatry, Charité Berlin, Germany.

^20^ Department of Psychiatry, University of Erlangen, Nürnberg, Germany.

^21^ LVR-Hospital Essen, Department of Psychiatry and Psychotherapy, University Duisburg-Essen, Germany.

^22^ Institute for Stroke and Dementia Reserach, Klinikum der Universität München, Marchioninistr. 15, 81377, Munich, Germany.

^23^ Department of Neurology, Klinikum der Universität München, Marchioninistr. 15, 81377, Munich, Germany.

^24^ Central Institute of Mental Health, Medical Faculty Mannheim, University of Heidelberg, Germany.

^25^ Department of Psychiatry, Psychosomatic Medicine and Psychotherapy, Goethe University, Frankfurt, Germany

^26^ Centre for Geriatric Medicine and Section of Gerontopsychiatry and Neuropsychology, Medical School, University of Freiburg, Germany.

^27^ Alzheimer Memorial Center and Geriatric Psychiatry Branch, Department of Psychiatry, Ludwig-Maximilian University, Munich, Germany

^28^ Departments of Psychiatry, Neurology and Genetics, Washington University School of Medicine, St Louis, MO 63110, US.

^29^ Department of Biology, Brigham Young University, Provo, UT, 84602, USA.

^30^ Department of Mental Health Sciences, University College London, UK.

^31^ The Wellcome Trust Sanger Institute, Wellcome Trust Genome Campus, Hinxton, Cambridge, UK.

^32^ Department of Genomics, Life & Brain Center, University of Bonn, Bonn, Germany

^33^ Department of Molecular Neuroscience, Institute of Neurology, University College London, Queen Square, London WC1N 3BG, UK

^34^ Deutsches Zentrum für Neurodegenerative Erkrankungen (DZNE), Bonn

**Information about the known variants:**

**Known pathogenic variant**

One known pathogenic mutation *PSEN1* p.A426P (rs63751223) was found. Rs63751223 was identified in five members of a family with early onset autosomal dominant AD [[1](#_ENREF_1),[2](#_ENREF_2)]. Interestingly, we found this variant in a non-demented (CDR=0) individual (56 years old) with low CSF tau levels (189.52 pg/ml, WU series) but relatively lower levels of CSF Aβ42 levels (653.69 pg/ml). No family history of dementia has been documented to date.

**Novel coding variants**

The novel variants are distributed among all the genes: A*PP* p.A741S and p.V287G, GRN p.C247Y, *MAPT* p.T263P, *PSEN1* p.V63G, and *PSEN2*, p.G270S, p.A346S, p.T347P and p.T369S (Table 2 and 3). Interestingly, the variants *APP* p.V287G, *PSEN1* p.V63G, and *PSEN2*, p.T347P and p.T369S were found in cognitively normal individuals with high Aβ42 CSF levels. On the other hand, the variant GRN p.C247Y was found in a 77 year-old individual with a CDR=0 but with low CSF Aβ42 levels (75 pg/ml, ADNI series) and high CSF ptau levels (35 pg/ml), which is considered biomarker criteria for AD [[3](#_ENREF_3)]. This variant is located in a highly conserved nucleotide (GERP=4.81); the residue p.C247 is conserved among all granulins and is very close related to a known pathogenic variant (p.P248L) reported in individuals with frontotemporal dementia (FTD) [[2](#_ENREF_2),[4](#_ENREF_4)].The variant *MAPT,* p.T263P was found in a cognitively normal individual (last CDR=0, 74 years old) with low CSF Aβ42 levels (406 pg/ml, WU series). This variant is located in exon 9 in a highly conserved nucleotide (GERP=5.41) and is predicted to be damaging for MAPT protein. Additionally, it falls within the first microtubule-binding domain of tau and is surrounded by known pathogenic mutations causing taupathies [[2](#_ENREF_2)]. The variant *PSEN2*, p.G270S was found in a demented individual (90 years old, CDR=0.5, MMSE= 24) with high CSF Aβ42 levels (247 pg/ml, ADNI series) and low CSF levels of ptau (19 pg/ml).

**Known low frequency variants**

We also identified four previously recognized high-risk variants for LOAD (*APOE*, p.L46P; *MAPT*, p.A152T; *PSEN2*, p.R62H and p.R71W) [[2](#_ENREF_2),[5](#_ENREF_5),[6](#_ENREF_6),[7](#_ENREF_7)]. *PSEN2* p.R71W was identified in one AD case (CDR=1, 82 years old) within the bottom 1% of CSF Aβ42 levels (81 pg/ml, ADNI series) and the top 18% of the CSF tau levels (141 pg/ml). *PSEN2* p.R71W exhibited a higher frequency in clinical cases than in controls (p=0.03, OR=10.3, 95%CI=1.1-96.2). However, it did not reach statistical significance in individuals with Aβ deposition (p=0.27, OR=3.4, 95%CI=0.38-30.7) (Table 3). *MAPT* p.A152T was found in one AD case (CDR=1, 84 years old) at the bottom 8% of CSF Aβ42 levels (140.3 pg/ml, ADNI series). It also occurred more frequently in clinical cases (MAF=0.006) than in controls (MAF=0.001), however, it did not achieve statistical significance in our dataset, possibly for the sample size (p=0.13, OR=3.9, 95%CI=0.64-23.3). *PSEN2* p.R62H was found in one AD case (CDR=0.5, 80 year old) within the bottom 9% of the CSF Aβ42 levels (273.41 pg/ml in the WU series) and *APOE* p.L46P was initially found in one AD case (CDR=1, 78 years old) with CSF Aβ42 levels within the bottom 7% (99.2 pg/ml, ADNI series). *PSEN2* p.R62H and *APOE* p.L46P carriers showed similar frequency among cases and controls (Table 3).

Seven variants that have been recently reported in public databases with no clear roles in human diseases to date were also found (*APOE*, p.E37K; *GRN*, p.C231W; *MAPT*, p.G107S, p.S318L, p.V224G; *PSEN2*, p.E317G and p.V300G) (Table 2). The variants *APOE* p.E37K, *MAPT* p.G107S and *PSEN2* p.V300G were found in cognitively normal individuals with high Aβ42 CSF levels (Table 3). In fact, *APOE* p.E37K was found in an individual (78 years old) within the top 1% of Aβ42 CSF levels (1211.68 pg/ml, WU series, *APOE* ε3/ ε3 genotype).

Six variants previously reported in families with AD or FTD but classified as non-pathogenic were also identified (*GRN*, p.R433W, p.P458L, p.R19W; *MAPT*, p.Q230R; *PSEN1*, p.R35Q and p.E318G) [[2](#_ENREF_2)]. The variant *GRN* p.P458L was found in a 37-year old individual with cognitive deficit (CDR=0.5), low Aβ42 CSF levels (241.6 pg/ml, WU series) and high tau CSF levels (924.09 pg/ml), which meets biomarker criteria for AD [[8](#_ENREF_8)]. *PSEN1* p.R35Q was found in a demented individual (Caucasian, 77 years old and CDR=0.5) who exhibits high Aβ42 CSF levels (221 pg/ml, ADNI series) and low CSF ptau levels (20 pg/ml), which suggest a non-AD type of dementia.

1. Poorkaj P, Sharma V, Anderson L, Nemens E, Alonso ME, et al. (1998) Missense mutations in the chromosome 14 familial Alzheimer's disease presenilin 1 gene. Hum Mutat 11: 216-221.

2. Cruts M, Theuns J, Van Broeckhoven C (2012) Locus-specific mutation databases for neurodegenerative brain diseases. Hum Mutat 33: 1340-1344.

3. Shaw LM, Vanderstichele H, Knapik-Czajka M, Clark CM, Aisen PS, et al. (2009) Cerebrospinal fluid biomarker signature in Alzheimer's disease neuroimaging initiative subjects. Ann Neurol 65: 403-413.

4. Guerreiro RJ, Washecka N, Hardy J, Singleton A (2010) A thorough assessment of benign genetic variability in GRN and MAPT. Hum Mutat 31: E1126-1140.

5. Scacchi R, Gambina G, Ferrari G, Corbo RM (2003) Screening of two mutations at exon 3 of the apolipoprotein E gene (sites 28 and 42) in a sample of patients with sporadic late-onset Alzheimer's disease. Neurobiol Aging 24: 339-343.

6. Cruchaga C, Haller G, Chakraverty S, Mayo K, Vallania FL, et al. (2012) Rare variants in APP, PSEN1 and PSEN2 increase risk for AD in late-onset Alzheimer's disease families. PLoS One 7: e31039.

7. Coppola G, Chinnathambi S, Lee JJ, Dombroski BA, Baker MC, et al. (2012) Evidence for a role of the rare p.A152T variant in MAPT in increasing the risk for FTD-spectrum and Alzheimer's diseases. Hum Mol Genet 21: 3500-3512.

8. Fagan AM, Head D, Shah AR, Marcus D, Mintun M, et al. (2009) Decreased cerebrospinal fluid Abeta(42) correlates with brain atrophy in cognitively normal elderly. Ann Neurol 65: 176-183.

| **Table S1: Biomarkers and covariates in the different series** | | |
| --- | --- | --- |
| **Pool1** | **vs Pool 2** | **vs Additional Set** |
| **Age** | ns | ns |
|  |  |  |
|  |  |  |
| **Gender** | ns | ns |
|  |  |  |
|  |  |  |
| **Apoe e4+** | ns | ns |
|  |  |  |
|  |  |  |
| **CDR = 0** | ns | ns |
|  |  |  |
|  |  |  |
| **Aß42** | <0.0001, * | <0.0001 δ |
|  |  |  |
|  |  |  |
| **Tau** | <0.0001 | <0.01 |
|  |  |  |
|  |  |  |
| **Ptau181** | <0.0001 | <0.01 |
|  |  |  |
|  |  |  |

| ANOVA was used to identify the important differences for each endophenotype, age and each set. The p-value shown. Fisher exact test was used for gender, apoe e4 + and CDR analysis, separate by series. ADNI pool1 vs pool2 p=0.01, δ ADNI pools vs additional data set is not significant |
| --- |

**Table S2. Summary of exon coverage per gene.**

| **GENE** | **Exon** | **Mean Coverage** |
| --- | --- | --- |
| **p53 positive control** |  | 335.9582 |
| **pCMV6-XL5 negative control** |  | 111.6862 |
| **APOE** | 1 | 196.4079 |
|  | 2 | 107.3219 |
|  | 3 | 82.1116 |
|  | 4 | 52.93052 |
| **GRN** | 1 | 158.054 |
|  | 2_4 | 175.2848 |
|  | 5_7 | 143.983 |
|  | 8_1 | 205.4358 |
|  | 11_13 | 207.851 |
| **PSEN1** | 1 | 221.6 |
|  | 2_3 | 77.77825 |
|  | 4 | 80.86913 |
|  | 5 | 95.76458 |
|  | 6 | 31.50099 |
|  | 7 | 54.82842 |
|  | 8 | 92.2601 |
|  | 9 | 85.52308 |
|  | 10 | 56.08312 |
|  | 11 | 64.90789 |
|  | 12 | 125.9575 |
| **PSEN2** | 1_2 | 16.47196 |
|  | 3 | 123.2815 |
|  | 4 | 140.2585 |
|  | 5 | 111.291 |
|  | 6 | 118.9234 |
|  | 7_8 | 112.3041 |
|  | 9 | 104.7415 |
|  | 10_11 | 141.1574 |
|  | 12 | 26.11573 |
|  | 13 | 74.70342 |

**Table S2. Summary of exon coverage per gene**

| **GENE** | **Exon** | **Mean Coverage** |
| --- | --- | --- |
| **APP** | 1 | 27.54009 |
|  | 2 | 197.2113 |
|  | 3 | 145.7021 |
|  | 4 | 94.60729 |
|  | 5 | 59.49297 |
|  | 6 | 130.5826 |
|  | 7 | 154.9414 |
|  | 8 | 98.93752 |
|  | 9 | 123.3836 |
|  | 10 | 62.32779 |
|  | 11 | 130.3605 |
|  | 12 | 124.6781 |
|  | 13 | 105.8592 |
|  | 14 | 97.83711 |
|  | 15 | 184.1279 |
|  | 16 | 118.9949 |
|  | 17 | 80.41112 |
|  | 18 | 64.74846 |
| **MAPT** | 1 | 61.95862 |
|  | 2 | 53.77114 |
|  | 3 | 81.24887 |
|  | 4 | 92.15595 |
|  | 5 | 62.5825 |
|  | 5 | 45.68207 |
|  | 7 | 47.82681 |
|  | 8 | 83.24144 |
|  | 9 | 79.76721 |
|  | 10 | 108.4224 |
|  | 11 | 96.46175 |
|  | 12 | 177.2816 |
|  | 13 | 137.7167 |
|  | 14 | 74.16285 |

**Table S2b. SPLINTER raw ouput of different SNPs by pools**

| **Pool 1** |  |  |  |  |  |  |
| --- | --- | --- | --- | --- | --- | --- |
| **Gene** | **Intronic** | **Missense** | **Coding-synonymous** | **Splicing** | **UTR** | **Near-gene** |
| **APOE** | 8 | 7 | 2 | 0 | 0 | 2 |
| **APP** | 62 | 2 | 2 | 0 | 18 | 0 |
| **GNR** | 13 | 3 | 2 | 0 | 4 | 0 |
| **MAPT** | 84 | 10 | 6 | 0 | 16 | 0 |
| **PSEN1** | 78 | 1 | 1 | 0 | 5 | 0 |
| **PSEN2** | 45 | 5 | 8 | 0 | 11 | 1 |
| **Total** | 290 | 28 | 21 | 0 | 54 | 3 |
|  |  |  |  |  |  |  |
| **Pool 2** |  |  |  |  |  |  |
| **Gene** | **Intronic** | **Missense** | **Coding-synonymous** | **Splicing** | **UTR** | **Near-gene** |
| **APOE** | 6 | 10 | 1 | 1 | 2 | 2 |
| **APP** | 56 | 1 | 1 | 0 | 4 | 0 |
| **GNR** | 8 | 3 | 1 | 2 | 2 | 1 |
| **MAPT** | 74 | 10 | 8 | 0 | 14 | 0 |
| **PSEN1** | 59 | 4 | 0 | 0 | 3 | 0 |
| **PSEN2** | 65 | 4 | 4 | 1 | 10 | 12 |
| **Total** | 268 | 32 | 15 | 4 | 35 | 15 |

SPLINTER software was used for call the SNPs. Here it is shown all the variants that passed the filter adjusting the sensitivity and specificity. Only variants with a predicted MAF less than 5 %, exonic missense and affecting the splicing, were selected to be validated.

**Table S3. Summary of sample Cerebrospinal Fluid (CSF) biomarker residual levels**

|  | **Sample** | **n** | **Residual_Aβ42 Mean ± SD (range)** | **Residual_Tau Mean ± SD (range)** | **Residual_p-tau Mean ± SD (range)** |
| --- | --- | --- | --- | --- | --- |
| **Pool 1** | **WU-ADRC** | 70 | 0.26 ± 0.38 (-0.53-1.02) | -0.21 ± 0.46 (-1.1-0.64) | -0.20 ± 0.41 (-1.08-1.27) |
|  | **ADNI** | 28 | -0.15 ± 0.31 (-0.83-0.24) | -0.43 ± 0.42 (-1.41-0.52) | -0.15 ± 0.31 (-0.83-0.24) |
| **Pool 2** | **WU-ADRC** | 75 | -0.32 ± 0.42 (-1.06-0.68) | 0.25 ± 0.59 (-1.33-1.25) | 0.24 ± 0.42 (-1.06-0.68) |
|  | **ADNI** | 39 | 0.04 ± 0.32 (-0.5-0.57) | 0.32 ± 0.58 (-0.97-1.21) | 0.39 ± 0.68 (-1.2-1.23) |
| **Additional Set** | **WU-ADRC** | 340 | 0.01 ± 0.31 (-1.2-0.78) | -0.009 ± 0.41 (-1.02-1.45) | -0.01 ± 0.37 (-0.86-1.42) |
|  | **ADNI** | 192 | 0.01 ± 0.21 (-0.69-0.57) | -0.003 ± 0.36 (-0.98-1.35) | -0.01 ± 0.34 (-0.87-1.08) |
| **Total** | **WU-ADRC** | **475** | 0.0002 ± 0.38 (-1.2-1.02) | 0.0014 ± 0.48 (-1.3-1.45) | 0.0015 ± 0.42 (-1.14-1.42) |
|  | **ADNI** | **259** | -1.930e-005 ± 0.25 (-0.83-0.57) | 3.862e-006 ± 0.47 (-1.14-1.35) | 7.721e-006 ± 0.45 (-1.58-1.23) |
